# The design and evaluation of hybrid controlled trials that leverage external data and randomization

**DOI:** 10.1038/s41467-022-33192-1

**Published:** 2022-10-02

**Authors:** Steffen Ventz, Sean Khozin, Bill Louv, Jacob Sands, Patrick Y. Wen, Rifaquat Rahman, Leah Comment, Brian M. Alexander, Lorenzo Trippa

**Affiliations:** 1 Division of Biostatistics, University of Minnesota, Minneapolis, MN USA; 2 CancerLinQ, New York, NY USA; 3 Project Data Sphere, Morrisville, NC USA; 4 Department of Medical Oncology, Dana-Farber Cancer Institute, Boston, MA USA; 5 Center for Neuro-Oncology, Dana-Farber Cancer Institute, Boston, MA USA; 6 Department of Radiation Oncology, Dana-Farber Cancer Institute, Boston, MA USA; 7 Foundation Medicine, Inc, Cambridge, MA USA; 8 Department of Data Science, Dana-Farber Cancer Institute, Boston, MA USA; 9 Department of Biostatistics, Harvard School of Public Health, Boston, MA USA

**Keywords:** Cancer, Public health, Cancer

## Abstract

Patient-level data from completed clinical studies or electronic health records can be used in the design and analysis of clinical trials. However, these external data can bias the evaluation of the experimental treatment when the statistical design does not appropriately account for potential confounders. In this work, we introduce a hybrid clinical trial design that combines the use of external control datasets and randomization to experimental and control arms, with the aim of producing efficient inference on the experimental treatment effects. Our analysis of the hybrid trial design includes scenarios where the distributions of measured and unmeasured prognostic patient characteristics differ across studies. Using simulations and datasets from clinical studies in extensive-stage small cell lung cancer and glioblastoma, we illustrate the potential advantages of hybrid trial designs compared to externally controlled trials and randomized trial designs.

## Abstract (web summary)

Patient-level external control data from prior clinical studies or electronic health records can be used in the design and analysis of clinical trials. Here the authors report a hybrid trial design combining the use of external control data and randomization to test experimental treatments, using small cell lung cancer and glioblastoma datasets as examples.

## Introduction

Randomized controlled trials (RCTs) are essential to demonstrate causal effects of an intervention on clinical outcomes. Randomization reduces the risk of bias by balancing potential confounders across treatment arms^1^. Though valuable, RCTs often require large samples sizes, resulting in long durations of accrual and high costs^2^. Non-randomized single-arm trials compare experimental treatments to historic benchmarks, and typically require smaller sample sizes than RCTs; however, they carry a risk of over- or underestimating treatment effects because of potential variations in patient populations across clinical trials^3–5^. The use of patient-level external control (EC) data from prior clinical studies has been proposed to reduce these risks and improve the evaluation of experimental treatments^6^.

The integration of EC data in the design and analysis of clinical trials can take several forms, including testing/estimating treatment effects upon study completion^5^, sample size re-estimation at interim analyses (IAs), and early decisions to terminate the study for futility or efficacy^7,8^. With the increasing availability of data from past trials, the prospective use of EC data in the design, conduct, and analysis of clinical trials has the potential to reduce the cost and time of evaluating new treatments^6,9,10^.

**Fig. 1:**
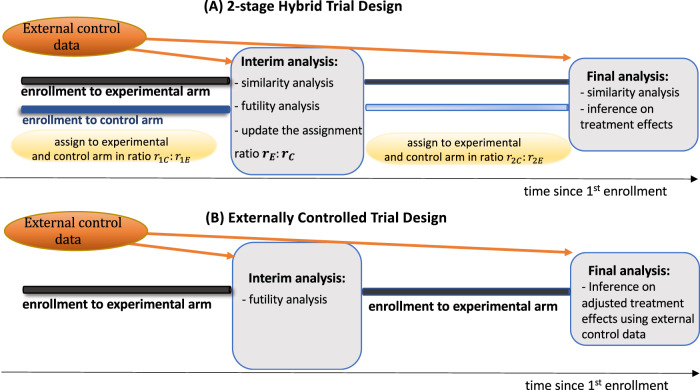
Two-stage hybrid trial (HT) and externally controlled trial (ECT) designs. Panel (**A**) shows a two-stage HT design, with *n*_1_ and *n*_2_ and enrollments to the internal control (IC) and experimental arm in ratios r_1E_:r_1C_ and r_2E_:r_2C_ during the first and second stages of the study, respectively. An interim analysis (IA) determines if the study is closed for futility or not, and potentially updates the randomization ratio from r_1E_:r_1C_ during the first stage to r_2,C_:r_2,E_ for the second stage of the study. These decisions are supported by an index of dissimilarity (see Methods) between the IC and external control (EC) populations. The same index of dissimilarity is recomputed at completion of the study and supports the decision to leverage the EC data for estimating the treatment effects of the experimental therapeutic or not. Panel (**B**) describes an ECT design that enrolls all *n* = *n*_1_ + *n*_2_ patients to the experimental arm. The ECT uses patient-level data of the experimental arm and external control data for a futility IA and for the estimation and testing (${{{{{{\rm{H}}}}}}}_{0}:{{{{{\rm{TE}}}}}}\le 0$) of treatment effects at the final analysis.

In this work, we introduce and examine a hybrid trial (HT) design that combines the use of EC data and randomization (Fig. 1) to test experimental therapeutics. We evaluate pivotal operating characteristics of the HT design such as power, the control of the false positive rates, and the average sample size and study duration. To evaluate these operating characteristics, we use simulations and two collections of datasets from clinical trials in newly diagnosed glioblastoma (GBM) and extensive-stage small cell lung cancer (ES-SCLC). We compare the HT design to single-arm externally controlled trials^5^ (ECTs), which leverage EC data, and RCTs. These comparisons illustrate the benefits, limitations, and risks of leveraging EC data using established metrics, such as the bias of treatment effects estimates and the average sample size.

## Results

We examined the operating characteristics of the HT design described in Methods. As summarized in Fig. 1, the first stage of the design randomizes ${{{{{{\rm{n}}}}}}}_{1}$patients to the experimental and internal control (IC) arms. The IA then determines if the study is closed for futility or not, and potentially updates the randomization ratio from 1:1 during the first stage of the study to r_2,C_:r_2,E_ for the second stage of the trial. These decisions are supported by an index of dissimilarity (see “Methods”) between the EC data and the early data from the IC arm. The same index of dissimilarity is recomputed at the completion of the study and supports the decision to leverage the EC data for estimating the treatment effects of the experimental therapeutic or not.

We compared HT, ECT^5^, and RCT designs using model-based simulations and in silico clinical trials generated with a resampling algorithm (see Methods) applied to ES-SCLC and GBM datasets.

### Model-based simulations

We considered a study with a maximum sample size of $120$ patients, an IA after $60$ enrollments, and a targeted type I error rate of ${{{{{\rm{\alpha }}}}}}=0.05$. ECTs and HTs utilized an EC dataset with 1000 patients. The size of the EC dataset is similar to the sample sizes of the ES-SCLC and GBM data collections. The simulated RCTs randomized all 120 patients to the IC and experimental treatment in a 1:1 ratio, while all 120 patients in the ECT received the experimental treatment.

Table 1 summarizes the simulation scenarios that we used to compare the study designs. To examine the robustness and illustrate potential pitfalls of the trial designs, we included scenarios (2–5) where relevant pre-treatment variables were not available for interim and final analyses. Moreover, in scenarios 4 and 5, the conditional outcome distributions of the IC and EC populations were different. Table 2 reports the results for each scenario, the average study duration, the average sample size, the proportion of trials that were terminated early for futility, and the type I error rate and power across 2000 (RCTs, ECTs, and HTs) simulations.

**Table 1:** Model-based simulation scenarios

Scenarios	Distribution of pre-treatment variables in the EC population			Effect of pre-treatment variables on the outcome in the EC (and HT) population			Response rates for the EC, IC, and EXPT		
	${p}_{1}$	${p}_{2}$	${p}_{3}$	${\theta }_{S,1}$	${\theta }_{S,2}$	${\theta }_{S,3}$	EC	IC and EXPT (TE = 0)	EXPT (TE > 0)
1	0.2	0.8	0.5	0.5	−0.5	0.0	0.43	0.50	0.68
2	0.2	0.8	0.1	0.5	−0.5	1.5	0.46	0.66	0.79
3	0.2	0.8	0.9	0.5	−0.5	1.5	0.73	0.66	0.79
4	0.2	0.8	0.1	0.5	−1.5(1.5)	1.5	0.30	0.66	0.79
5	0.2	0.8	0.9	0.5	−1.5(1.5)	1.5	0.55	0.66	0.79

We consider three binary pre-treatment variables *X* = (*X*_1_, *X*_2_, *X*_3_). The variable ${X}_{3}$ is not available and is not used in the interim and final analyses. For patients enrolled in the hybrid trial (HT), the three pre-treatment variables are independent, with ${p}(X_{j}=1)=0.5$ for $j={{{{\mathrm{1}}}}},\,{{{{\mathrm{2}}}}},\,{{{{\mathrm{3}}}}}.$ Columns 2–4 report the distribution ${p}(X_{j}=1)$ of the three independent variables in the external control (EC) population. Patient outcomes Y, given the pre-treatment variables, were randomly generated from a logistic model, $p\left(\right.Y=1\left|X,\,A,\,S\right)=F\left(\delta A+X^{\prime} {\theta }_{S}\right),\,{A}={{{{\mathrm{0,\,1}}}}}$ and $S={{{{\mathrm{0,\,1}}}}},$ where $F\left(t\right)=1/(1+{{\exp }}\{-t\})$. Columns 5–7 show the effects (${\theta }_{S,j}$, log odds ratio) of the pre-treatment variables ${X}_{j}$ on the expected outcome Y in the EC (*S* = 1) and HT (*S* = 0) populations. When ${\theta }_{0,j}={\theta }_{1,j}$ we omit the value in parenthesis (${\theta }_{0,j}$). The treatment effect (TE, log odds ratio) for ineffective and effective experimental treatments equals $\delta=0,\, 0.8$. Columns 8–10 show the average response probability for the EC $(A=0,\, S=1)$, the internal control (IC) $(A=0,\, S=0)$, and the experimental treatment (EXPT, $A=1,\, S=0$) populations with and without treatment effects.

**Table 2:** Operating characteristics of the HT, ECT and RCT designs

	No treatment effect (TE=0)					Positive treatment effect (TE > 0)				
Design	HT	HT	HT	ECT	RCT	HT	HT	HT	ECT	RCT
randomization ratio ${r}_{2,C}:{r}_{2,E}$	1:1	1:2	0:1	0:1	1:1	1:1	1:2	0:1	0:1	1:1
*Scenario 1: No unmeasured confounding*, $P\left(Y=1|A=0,\,S=1\right) \, > \, P\left(Y=1|A=0,\,S=0\right)$										
Type I error rate (%)	6	4	6	4	5	–	–	–	–	–
Power (%)	–	–	–	–	–	70	71	73	93	67
% of trials stopped at IA	20	15	15	44	7	0	0	0	0	0
Average study duration	21	22	22	18	23	24	24	24	24	24
Average sample size	108	112	111	93	115	120	120	120	120	120
*Scenario 2: Unmeasured confounding*, $P\left(Y=1|A=0,\,S=1\right) \, > \, P\left(Y=1|A=0,\,S=0\right)$										
Type I error rate (%)	6	7	8	71	5	–	–	–	–	–
Power (%)	–	–	–	–	–	54	55	56	100	54
% of trials stopped at IA	8	8	8	2	7	0	0	0	0	0
Average study duration	23	23	23	24	23	24	24	24	24	24
Average sample size	115	115	115	119	115	120	120	120	120	120
*Scenario 3: Unmeasured confounding*, $P\left(Y=1|A=0,\,S=1\right) \, < \, P\left(Y=1|A=0,\,S=0\right)$										
Type I error rate (%)	5	5	6	0	5	–	–	–	–	–
Power (%)	–	–	–	–	–	54	53	54	12	53
% of trials stopped at IA	18	12	12	98	7	4	3	3	42	0
Average study duration	22	22	22	12	23	23	23	23	19	24
Average sample size	109	113	113	61	116	118	118	118	95	120
*Scenario 4: Unmeasured confounding*, $P\left(Y=1|A=0,\,S=1\right) \, > \, P\left(Y=1|A=0,\,S=0\right)$										
Type I error rate (%)	5	5	6	>99	5	–	–	–	–	–
Power (%)	–	–	–	–	–	53	53	53	100	54
% of trials stopped at IA	7	7	8	0	7	1	0	1	0	1
Average study duration	23	23	23	24	23	24	24	24	24	24
Average sample size	116	116	115	120	116	120	120	120	120	120
*Scenario 5: Unmeasured confounding*, $P\left(Y=1|A=0,\,S=1\right) \, > \, P\left(Y=1|A=0,\,S=0\right)$										
Type I error rate (%)	6	7	9	15	5	–	–	–	–	–
Power (%)	–	–	–	–	–	65	65	65	93	53
% of trials stopped at IA	22	15	15	55	7	1	1	1	1	1
Average study duration	21	22	22	17	23	24	24	24	24	24
Average sample size	107	111	111	87	115	119	119	119	119	120

We consider different distributions of measured (${X}_{1},\,{X}_{2}$) and unmeasured (${X}_{3}$) patient pre-treatment characteristics (see Table 1 for details). We provide results for an experimental treatment with (columns 7–11) and without (columns 2–6) positive treatment effects (TEs). For each scenario, we report the type I error rate (i.e., the probability of rejecting the null hypothesis when TE = 0), the power (i.e., the probability of rejecting the null hypothesis when TE > 0), the proportion of trials stopped early for futility, the average sample size, and average study duration (months) across 2000 simulations.

Scenario 1 (defined in Table 1), where all relevant pre-treatment patient characteristics are available for analysis, represents an ideal condition for leveraging EC data. Here, all designs have type I error rates close to the targeted 5% level (see Table 2). As expected, the ECT has superior performance compared to HTs and RCTs. For instance, without a positive treatment effect, 44% of ECTs were terminated early for futility, compared to 7% and 15–20% for RCTs and HTs, respectively. In scenario 1, the RCT had approximately 67% power, compared to 93% and 70–73% for the ECT and HT designs.

In scenarios 2–5, the set of available prognostic pre-treatment variables for the interim and final analyses is incomplete and statistical assumptions for inference in ECTs are therefore violated. In these scenarios, the ECT design performed worse than the HT and RCT designs. For instance, in scenarios 2 and 4, without a positive treatment effect, 71% (>99%) of the generated ECTs reported a false positive result (type I error), compared to 5–8% for the HT design and 5% for the RCT design. Moreover, in scenario 3, the power of the ECT design declined to 12% compared to $\ge$53% for the RCT and HT designs.

We also compared HT, ECT and RCT designs when the experimental treatment is inferior to the SOC (TE < 0, see Tables S9 and S10). Similar to the scenarios without treatment effects (TE = 0, rows 2–6 in Table 2), the HT design reduces the type I error rate compared to the ECT if there is confounding (Supplementary Table 9, scenarios 4 and 6). Moreover, when TE < 0, the HT design terminates the study early for futility with higher probability than the RCT design.

### In silico trials in ES-SCLC

We performed a literature review and identified pre-treatment characteristics associated with overall survival (OS) in ES-SCLC (column 1 of Supplementary Table 1). Only three of these variables (sex, age, and ECOG performance status) were available in the datasets (CALGB-9732^11^, GALES^12^ and Pirker et al.^13^) and were included in our analyses (Supplementary Table 1).

The effects of pre-treatment variables on OS were estimated for patients treated with the standard of care (SOC) using a Cox model^14^, with baseline survival stratified by studies (Supplementary Table 3). Sex (male vs female, HR 1.45, *p* < 0.001), age (<65 years vs ≥65, HR 0.7, *p* < 0.001), and performance status (1 vs 0 HR 1.28, *p* = 0.024, 2 vs 0 HR 2.54, *p* < 0.001) had a significant association with OS. To investigate heterogeneity across studies, we estimated study-specific random effects in a Cox model for OS (column 3 of Supplementary Table 3). These random effects represent differences of the outcome distributions across trial populations that are not attributed to the available patient pre-treatment characteristics. The estimates suggest differences in the conditional outcome distributions (i.e., given the available pre-treatment variables) between studies. The limited availability of pre-treatment patient characteristics, as well as the random effects analyses, indicate limitations of the ES-SCLC datasets as EC for future ES-SCLC trials.

We considered a study with a size of 75 patients and OS at 9 months (OS-9) as primary endpoint. For the HT design, 50 and 25 patients were enrolled during the first stage (1:1 randomization) and second stage (r_2,C_:r_2,E_ equal to 0:1), respectively. We report results for additional values of the design parameters in the Supplementary Information. We used block randomization; for example, for RCTs, 12 patients per arm (experimental and control) were assigned during the second stage (25 patients) and the last patient was randomly assigned.

**Fig. 2:**
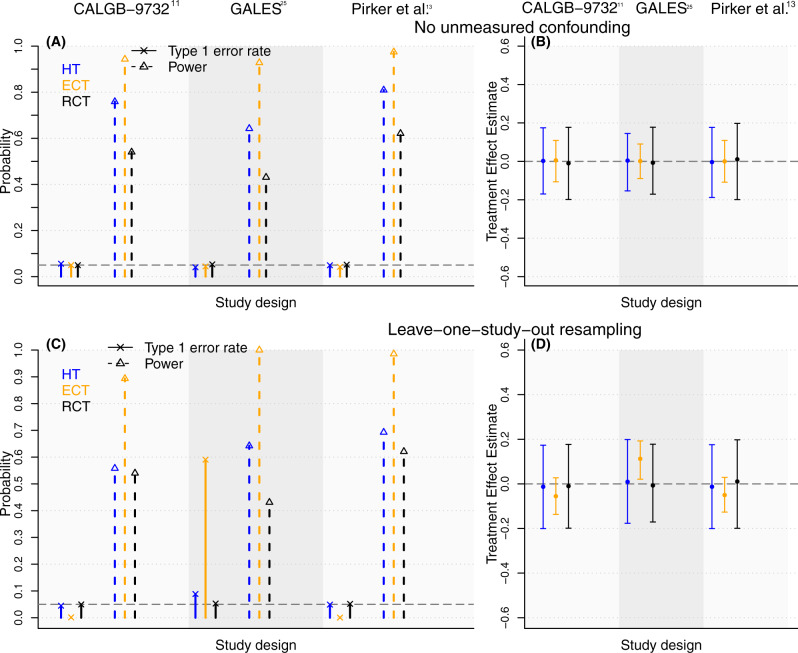
Operating characteristics of in silico HTs, ECTs and RCTs generated by resampling the control arms of the ES-SCLC studies. The top row shows type I error rates (panel **A**, solid vertical lines with a cross), power (Panel **A**, dotted vertical lines with an arrow), and the variability/bias of the treatment effect estimates (panel **B**). In panel (**B**), the dots indicate the average treatment effect estimates across in silico trials (*n* = 75) and the vertical bars indicate the 5% and 95% quantiles. Panels (**A**) and (**B**) are representative of an ideal setting, without unmeasured confounders, and identical conditional outcome distributions of the SOC across studies. The bottom row (Panels **C** and **D**) shows the same operating characteristics as the top row when we used the leave-one-study-out resampling algorithm to generate in silico trials (*n* = 75).

Figure 2 shows selected characteristics of the ECT, HT, and RCT designs based on 2000 resampled trials. The resampling algorithm to generate these in silico trials is described in Methods. The bottom row of Fig. 2 illustrates the operating characteristics when we apply the resampling algorithm. Each panel includes three columns that indicate the study (CALGB-9732^11^, GALES^12^ and Pirker et al.^13^) that was resampled to generate in silico trials. The results reflect the underlying study-to-study heterogeneity and the described limitations of the ES-SCLC datasets.

The top row of Fig. 2 illustrates the same operating characteristics of the three trial designs under an ideal setting, without unmeasured confounders and differences of the conditional outcome distributions under the control treatment across studies. This was achieved by first randomly permuting the study membership labels of patients in the ES-SCLC datasets and then applying the resampling algorithm. These results serve as a reference to illustrate differences between the operating characteristics of ECTs and HTs under ideal settings for leveraging EC data (top row) and with the actual study-to-study differences (bottom row) in the ES-SCLC datasets.

Panels A and C of Fig. 2 show the estimated type I error rates (solid vertical lines; target value 5%) and power (dotted vertical lines) of the HT, ECT, and RCT designs when we resampled the CALGB-9732^11^, GALES^12^, and Pirker et al.^13^ studies. As expected, without cofounding (Fig. 2A), the ECT was the most powerful design, with 94%, 97%, and 93% power for the CALGB-9732, Pirker et al., and GALES studies, respectively, compared to 76%, 80%, and 65%, and 54%, 62%, and 43% for the HT and RCT designs in each of the three studies, respectively. In contrast, because of study-to-study heterogeneity, the resampling algorithm (Fig. 2C) showed that the ECT design inflates the type I error rates, which reaches 59% for the GALES study. The type I error rates were considerably lower for the HT design (5%, 8%, and 5% for CALGB-9732^11^, GALES^12^ and Pirker et al.^13^), as the dissimilarity analyses (see “Methods”) recognize the limitations of the EC data.

We also compared the HT, ECT, and RCT designs for clinical trials with overall survival as primary outcome (see Section S1.5 of the Supplementary Information for statistical details). In particular, Supplementary Fig. 5 illustrates operating characteristics of in silico HTs, ECTs and RCTs that we generated applying the model-free resampling algorithm to the CALGB-9732^11^, GALES^12^, and Pirker et al.^13^ datasets. Similar to the results for in silico trials with binary outcomes (Fig. 2), the type I error rates of the ECT design deviate substantially from the nominal 5% level, i.e. <1% for the CALBG-9732 and Piker et al. studies, and 14% for the GALES study. In contrast, for the HT, we observed type I error rates very close to the nominal 5% level (5.0%, 5.2%, and 4.9%).

### In silico trials in GBM

We used five GBM^5,15^ datasets (see Methods and Supplementary Table 5) to compare HT, ECT, and RCT designs. We considered a study with a sample size of 100 patients, OS-12 as the primary endpoint, and an IA after 50 enrollments. The initial randomization ratio r_1,C_:r_1,E_ was 1:1 for both the HT and RCT designs. For the HT design, the randomization ratio during the second stage remained 1:1 or was updated to r_2,C_:r_2,E_ (we considered 1:2 and 0:1). We generated in silico trials resampling from GBM datasets (Chinot et al.^16^, Dana-Farber Cancer Institute^5^ [DFCI], and University of California, Los Angeles^5^ [UCLA]) with more than 100 patients treated with the current SOC^17^, temozolomide in combination with radiation therapy.

In contrast to ES-SCLC, the GBM datasets included all major prognostic patient pre-treatment characteristics identified through a literature review^5^. This difference between the ES-SCLC and GBM datasets is consistent with results obtained from Cox regression models with study-specific random effects (Supplementary Table 6). The estimated model indicates lower study-to-study variability in the GBM datasets compared to the ES-SCLC datasets.

Table 3 shows selected operating characteristics of the ECT, HT, and RCT designs based on 2000 in silico trials generated by resampling the SOC arms of the Chinot et al. (rows 4–10 and 26–32), DFCI (rows 11–17 and 33–39), and UCLA (rows 18–24 and 40–46) datasets. Rows 4–24 (26–46) correspond to in silico RCTs, ECTs, and HTs that evaluated an experimental treatment with (or without) a positive treatment effect.

**Table 3:** Resampling-based evaluation of the operating characteristics of the HT, ECT, and RCT designs in GBM

Design	HT	HT	HT	ECT	RCT
Assignment ratio *r*_2,*C*_:r_2,*E*_	1:1	1:2	0:1	0:1	1:1
**No treatment effect (TE** **=** **0)**					
Chinot et al.^16^					
Type I error rate (%)	5	6	5	3	5
% of trials stopped at IA	24	24	24	42	7
Average study duration	17	17	17	16	19
Average sample size	88	88	88	78	96
Average TE estimate	−0.02	−0.02	−0.02	−0.01	−0.01
(10% and 90% quantiles)	(−0.16,0.12)	(−0.15,0.12)	(−0.14,0.10)	(−0.09,0.07)	(−0.16,0.12)
DFCI^5^					
Type I error rate (%)	6	5	4	3	6
% of trials stopped at IA	27	27	25	50	7
Average study duration	17	17	17	15	19
Average sample size	86	87	88	75	96
Average TE estimate(10% and 90% quantiles)	−0.02 (−0.16,0.12)	−0.02 (−0.15,0.11)	−0.01 (−0.14,0.10)	−0.02 (−0.09,0.07)	−0.01 (−0.16,0.12)
UCLA^5^					
Type I error rate (%)	5	6	5	3	4
% of trials stopped at IA	25	24	24	42	6
Average study duration	17	17	17	16	19
Average sample size	88	88	88	79	97
Average TE estimate	−0.02	−0.02	−0.02	−0.02	−0.01
(10% and 90% quantiles)	(−0.14,0.12)	(−0.15,0.11)	(−0.14,0.09)	(−0.09,0.06)	(−0.14,0.12)
**Positive treatment effect (TE > 0)**					
Chinot et al.^16^					
Power (%)	73	78	84	85	58
% of trials stopped at IA	<1	<1	<1	<1	<1
Average study duration	20	20	20	20	20
Average sample size	100	100	100	100	100
Average TE estimate	0.15	0.15	0.15	0.15	0.15
(10% and 90% quantiles)	(0.04,0.25)	(0.05,0.25)	(0.05,0.24)	(0.09,0.21)	(0.06,0.26)
DFCI^5^					
Power (%)	77	82	88	92	63
% of trials stopped at IA	<1	<1	<1	<1	<1
Average study duration	20	20	20	20	20
Average sample size	100	100	100	100	100
Average TE estimate	0.17	0.17	0.17	0.18	0.17
(10% and 90% quantiles)	(0.04,0.3)	(0.06, 0.27)	(0.08,0.26)	(0.11,0.24)	(0.06,0.28)
UCLA^5^					
Power (%)	73	78	85	86	58
% of trials stopped at IA	<1	1	<1	<1	<1
Average study duration	20	20	20	20	20
Average sample size	100	100	100	100	100
Average TE estimate	0.15	0.15	0.15	0.14	0.15
(10% and 90% quantiles)	(0.04,0.26)	(0.04,0.25)	(0.05,0.24)	(0.08,0.21)	(0.04,0.26)

We used individual-level data from patients treated with TMZ+RT from five GBM datasets. Rows 3–24 and 25–46 show results for an experimental treatment without a treatment effect (TE, rows 3–24) and with a positive TE (rows 25–46), respectively. We report the type I error rate (i.e., the probability of rejecting the null hypothesis when TE = 0), the power (i.e. the probability of rejecting the null hypothesis when TE > 0), the proportion of trials stopped early for futility, the average sample size, the average study duration (months), and the average (10% and 90% quantiles) estimate of the treatment effect, ${{{{{{\rm{TE}}}}}}}=E\left[p\left(Y=1|X,\,A=1\right)-p\left(Y=1|X,\,A=0\right)\right]$ across 2000 in silico trials.

All three study designs showed type I error rates across in silico trials close to the targeted 5% level. Both the ECT and HT designs had a higher probability (42–50% for ECTs and 24–27% for the HTs) of stopping the study early when the treatment effect was null compared to the RCT design (6–7%). This translates into reductions of the average sample size of the in silico ECTs and HTs compared to the RCTs, from 96 patients for the RCT design to 75–79 patients and 86–88 patients for the ECT and HT designs. Moreover, for the in silico GBM trials that evaluated an effective experimental treatment (rows 26–46), we observed gains in power for ECT (85–92%) and HT (73–77%, 78–82%, and 74–78% with r_2,C_:r_2,E_ equal to 1:1, 1:2, and 0:1, respectively) designs compared to conventional RCTs (58–63%).

## Discussion

The increasing availability of patient-level data from completed clinical studies and electronic health records constitutes an opportunity for the development of novel trial designs that leverage EC data^5,7,9,15,18^. Recent contributions^5,8,18^ have proposed methodologies to integrate EC data into the analysis of single-arm trials (ECTs). These methods replace published estimates of the SOC’s efficacy used as a benchmark with patient-level EC data. The EC data in ECTs allow the analyst to account for variations in the distribution of prognostic pre-treatment characteristics across clinical studies. This approach has the potential to reduce bias, false positive/negative rates, and ultimately improve the evaluation of experimental treatments^4,5^.

As illustrated in recent retrospective studies^5,19,20^ and in Table 2, under ideal conditions—without unmeasured confounding and with moderate variations of the patient pre-treatment profiles across study populations—the ECT design is an attractive alternative to the RCT design. However, it is challenging to anticipate mechanisms, such as unmeasured confounding and variations of the trial population during the enrollment period, which can bias the primary findings of the study.

Statistical methods applicable to ECTs, such as marginal structural models (MSMs)^21^, matching^22^, and inverse-probability weighting^23^ (IPW), rely on key assumptions that are difficult to validate. They assume that (a) all confounding pre-treatment variables are available and included in the analyses; (b) consistent definitions and standards are used to measure patient profiles and outcomes during the trial and in the EC; and (c) identical conditional outcome distributions, given the patient pre-treatment characteristics, under the control therapy for the EC and the trial population. If these assumptions are violated, then the treatment effects estimate can be biased, and the control of false positive rates can be compromised (see Table 2 and Fig. 2).

During the design phase of an ECT, it is challenging to quantify the risks associated with leveraging EC data. For example, unexpected confounding variables may not be included in the EC data, or subtle differences in the definition or measurement standards of the patient characteristics and treatment outcomes may remain unnoticed. Importantly, the data generated during the trial do not provide evidence in favor or against the ECT assumptions, as the study does not have a control arm.

In consideration of these challenges, we introduced a hybrid design that combines randomization and the use of EC data. We developed the design to achieve and balance two goals. First, we aimed for reliable inference of the treatment effects even in settings where the EC data have limitations. This included unmeasured confounding and other mechanisms that translate into poor operating characteristics of ECTs (see Table 2 and Fig. 2). Second, we sought to achieve efficiency levels comparable to ECTs in the ideal setting, when the EC data have no limitations and the ECT assumptions hold. In these scenarios, it is convenient to leverage the EC data to improve the trade-off between power and the resources for conducting the trial (Table 3).

In settings where discrepancies between the conditional outcome distributions of the EC group and the control arm are likely to occur, both the HT and ECT designs are not applicable. If multiple EC datasets are available, then meta-analyses and resampling algorithms (see “Methods” section) can be used to scrutinize the EC data and detect confounding^5,8,15^. Nonetheless, potential pitfalls associated with the use of EC data in a future study cannot be ruled out. These risks include potential unmeasured differences between the patients that will be enrolled and the EC group, as well as overlooked incongruences in the definitions of the outcomes^5^. For example, discrepancies between patient imaging schedules in the trial and the EC group correlate with the assessment of progression free survival outcomes and can introduce confounding.

A major difference between ECTs and HTs is the use of prospective dissimilarity analyses to attenuate the outlined risks. HTs evaluate if there is evidence of differences between the conditional outcome distributions in the EC group and in the control arm of the study. The EC data are used for inference on the treatment effects only if the resulting index of dissimilarity does not suggest different conditional distributions. The dissimilarity thresholds of the HT design can be tuned using simulations, to balance the trade-off between (i) leveraging EC data in settings without confounding mechanisms and (ii) the goal of controlling the risk of bias and inflated false positive or negative rates.

The integration of EC data and the proposed HT design can increase the power of the study. For example, consider a clinical trial with binary outcomes and an overall sample size of 100 patients. The response probabilities for the SOC and the experimental treatment are 0.6 and 0.78. An RCT with 1:1 randomization, which controls the type I error rate at the 5% level, has 62% power. We compare the RCT to a HT design with an EC group of 1000 patients and 1:1 randomization for the first 50 enrolled patients. The randomization changes to 1:2 (or 1:3) for the next 50 patients if ${{{{{{\rm{W}}}}}}}_{1} \, < \, {{{{{{\rm{w}}}}}}}_{1}$ (see “Methods” for the definition of the dissimilarity indices ${{{{{{\rm{W}}}}}}}_{1}$ and ${{{{{{\rm{W}}}}}}}_{2}$). Assume for simplicity that there are no relevant pre-treatment variables or other confounding mechanisms. The HT design has 90% conditional power when the dissimilarity summaries don’t exceed the dissimilarity thresholds and therefore the EC data are used in the final analyses. Here the conditional power indicates the probability of rejecting the null hypothesis given ${{{{{{\rm{W}}}}}}}_{1} \, < \, {{{{{{\rm{w}}}}}}}_{1}$ and ${{{{{{\rm{W}}}}}}}_{2} \, < \, {{{{{{\rm{w}}}}}}}_{2}$. In the outlined example, when we focus on HTs in which randomization changed to 1:2 (or 1:3) during the 2nd stage of the HT, but the final analyses don’t include the EC data (i.e., ${{{{{{\rm{W}}}}}}}_{1} \, < \, {{{{{{\rm{w}}}}}}}_{1}$ and ${{{{{{\rm{W}}}}}}}_{2}\ge {{{{{{\rm{w}}}}}}}_{2}$), the conditional power (61.7% and 60.9%) remains similar to the power of the RCT (62%).

For the proposed HT design, if randomization is updated during the second stage (i.e. ${{{{{{\rm{W}}}}}}}_{1} \, < \, {{{{{{\rm{w}}}}}}}_{1}$), but the dissimilarity index at the final analysis exceeds the threshold, then the conditional power (given ${{{{{{\rm{W}}}}}}}_{1} \, < \, {{{{{{\rm{w}}}}}}}_{1}$ and ${{{{{{\rm{W}}}}}}}_{2}\ge {{{{{{\rm{w}}}}}}}_{2}$) of the study may be below the targeted overall power level, say 80%. In our previous example, a randomization ratio r_C,2_:r_E,2_ of 1:2 (or 1:3) led to minor reductions (<2% points) in conditional power compared to the power of an RCT with identical sample size. But a ratio r_C,2_:r_E,2_ of 0:1 would reduce the conditional power of the HT by approximately 13% compared to the RCT. We can consider two strategies to address this potential limitation. First, the HT design can include a sample size extension, and enroll an additional group of ${{{{{{\rm{N}}}}}}}_{3}$ patients after the 2nd dissimilarity and futility IA (when ${{{{{{\rm{W}}}}}}}_{1} \, < \, {{{{{{\rm{w}}}}}}}_{1}$ and ${{{{{{\rm{W}}}}}}}_{2}\ge {{{{{{\rm{w}}}}}}}_{2}$). In this case the futility IA avoids a sample size extension if the data are not promising. The sample size ${{{{{{\rm{N}}}}}}}_{3}$ can be selected to ensure a conditional power of 80% (when ${{{{{{\rm{W}}}}}}}_{1} \, < \, {{{{{{\rm{w}}}}}}}_{1}$ and ${{{{{{\rm{W}}}}}}}_{2}\ge {{{{{{\rm{w}}}}}}}_{2}$). The second solution consists in selecting the overall sample size of the HT and the randomization ratios to ensure that the conditional power does not drop below a prespecified minimum, say 77% (i.e., we accept a reduction of ≤3% conditional power compared to the targeted overall power of 80%) when the final analysis of the HT excludes the EC data, ${{{{{{\rm{W}}}}}}}_{1} \, < \, {{{{{{\rm{w}}}}}}}_{1}$ and ${{{{{{\rm{W}}}}}}}_{2}\ge {{{{{{\rm{w}}}}}}}_{2}$.

The integration of EC data in HTs can improve interim decisions. For example, we used data from the experimental and control arms of the Pirker et al.^13^ study and conducted retrospective analyses to evaluate the likelihood of terminating the study early for futility using either an RCT design or a HT design. The reported OS Kaplan-Meier curves and the median OS (approximately 40 weeks) were nearly identical for the experimental and control arms of the study. We considered RCT and HT designs with an overall sample size of 100 patients, OS-9 primary outcome, and an IA after the outcomes of the first 50 enrolled patients become available. We used a resampling algorithm that is nearly identical to the one used in the Results Section. The HT design, leveraging EC data, stopped 57% of in silico trials for futility at the IA. In comparison, 19% of the in silico RCT (without using EC data) were stopped at the IA.

We used datasets from completed clinical studies and electronic health records to create realistic scenarios that highlight potential risks and benefits of the ECT and HT designs. ES-SCLC and GBM datasets were used to compare HT, ECT, and RCT designs. The scenarios defined by resampling the control arms of the ES-SCLC datasets are representative of settings where ECTs have poor operating characteristics due to confounding. Scenarios defined through GBM datasets were markedly different. In the resulting in silico GBM trials, leveraging EC data translated into efficiency gains compared to RCTs while maintaining control of false positive rates. The analyses based on model-based simulations (Table 2) and in silico trials obtained by resampling the GBM datasets^8^ (Table 3) indicated potential efficiency gains of HTs compared to RCTs when EC data without substantial limitations are available. We showed improvements of power, average study duration, and sample size.

A limitation of our analyses is the relatively small number of GBM and ES-SCLC datasets used to evaluate the HT and ECT designs. A larger number of datasets could provide a more representative sample of outcome distributions and other important differences across SOC arms of recent RCTs in GBM and ES-SCLC. Moreover, only a small subset of known prognostic pre-treatment variables (Supplementary Table 1) was available in the ES-SCLC datasets for statistical adjustments in ECTs and HTs. One study was open label (GALES^24^) and another one was only partially randomized (CALGB-30504^25^). Additionally, there were variations of the eligibility criteria across the ES-SLCLC studies, and etoposide with either platinum-based cisplatin or carboplatin chemotherapy were two SOC regimens in these trials. With these data limitations, the type I error rate of the ECT design in ES-SCLC, accounting for a limited set of available prognostic variables (Supplementary Table 1), was as high as 59% in our analyses.

When there is uncertainty regarding the risks associated with available EC data, the proposed HT design can be an attractive alternative to the ECT and RCT designs. Limitations of the EC data can impact the operating characteristics of ECTs, while at the opposite end of the spectrum RCTs do not utilize EC data. HTs can be viewed as a compromise between ECTs and RCTs, as HTs prospectively evaluate potential limitations of the EC data which are compared to the IC arm.

The described limitations of the datasets (e.g., different eligibility criteria), the random effects analysis (Supplementary Table 3), and the in silico ECTs (Fig. 2) consistently associated the use of the ES-SCLC datasets to specify an EC group with risks of bias and inadequate control of false positive/negative rates. We used the ES-SCLC datasets primarily to illustrate that HTs could substantially reduce these risks compared to ECTs.

ECTs have been considered previously in settings beyond ES-SCLC and GBM. Carrigan et al.^19^ demonstrated the feasibility of generating external controls in non-small cell lung cancer (NSCLC) using real-world data from the Flatiron Health database. Similarly, in Project Switch^20^, FDA investigators showed that ECTs can estimate OS hazard ratios by exchanging the control arms between trials in second-line NSCLC with docetaxel controls.

The integration of EC data into clinical trials requires high-quality and up-to-date patient-level datasets representative of the current SOC. Factors such as changes in the SOC and the discovery of new prognostic biomarkers pose challenges in maintaining contemporaneous EC datasets. On the other hand, HTs and EC data with biomarker information can be useful for testing novel treatments in subpopulations with low enrollment rates. Moreover, HT designs can be extended to alternative study aims, such as testing non-interiority. Recent data sharing efforts^26^, such as the National Cancer Institute (NCI) NCTN/NCORP Data Archive, Project Data Sphere^27^, YODA^28^, Vilvi^29^, and CancerLinQ^30^, provide valuable data sources for this endeavor.

## Methods

The research complied with ethical regulations and was approved by an institutional review board at DFCI.

We use $Y$ to indicate the binary primary outcome. We also report results for time-to-event primary endpoints $Y$ (e.g., OS) in the Supplementary Information. The binary variable $A$ indicates whether the patient received the experimental ($A$ = 1) or control ($A=0$) therapy, and the vector **X** includes a fixed set of pre-treatment patient characteristics (e.g., age, sex, etc.). The indicator S distinguishes patients enrolled during the trial ($S=0$) from patients in the external control (EC) dataset ($S=1$). Patients in the EC group were treated with the control therapy ($A=0$). We use ${\Pr }\left(Y|{{{{{\mathbf{X}}}}}},\,A,\,S\right)$ to indicate the conditional outcome distribution of patients with pre-treatment characteristics ${{{{{\mathbf{X}}}}}}$ and treatment $A$in the trial population ($S=0$) or in the EC group ($S=1$).

### Hybrid design

Figure 1A describes a HT design that uses EC data and randomization to the experimental and control (internal control, IC) arms to estimate and test treatment effects. For simplicity, we focused on a two-stage design with sample size ${n=n}_{1}+{n}_{2}$. During the first stage ${n}_{1}$ patients are randomized to the IC and experimental arms in the ratio r_1,C_:r_1,E_ (1:1 in our analyses). At completion of the first stage, after enrollment of the first *n*_1_ patients, an IA is used to decide (a) if the clinical study continues to the second stage or is stopped for futility; and, if the study is not stopped for futility, (b) whether or not to update the randomization ratio to r_2,C_:r_2,E_ for the remaining ${{{{{{\rm{n}}}}}}}_{2}$patients during the second stage. These two decisions are supported by an index of dissimilarity (${{{{{{\rm{W}}}}}}}_{1}$, Supplementary Information), computed using early data from the trial and the EC dataset. The summary ${{{{{{\rm{W}}}}}}}_{1}$ quantifies the evidence of differences between the conditional outcome distributions ${\Pr }\left(Y|{{{{{\mathbf{X}}}}}},\,A=0,\,S\right)$of the IC ($S=0$) and EC ($S=1$) populations. Large values of ${W}_{1}$ indicate dissimilarity between the two conditional distributions. In particular,

- if ${W}_{1}$ exceeds a predefined threshold ${w}_{1}$ (${W}_{1} \, > \, {w}_{1}$), then the EC data are excluded from the futility analysis and, if the trial is not stopped for futility, the assignment ratio during the second stage remains 1:1, as in the first stage.
- If ${W}_{1}\le {w}_{1}$, then the futility IA utilizes both IC and EC data. If the trial is not stopped for futility, the proportion of patients assigned to the IC during the second stage is decreased by updating the assignment ratio to the prespecified value r_2,C_:r_2,E_ . We considered ratios of 1:1, 1:2, and 0:1. When r_2,C_:r_2,E_ = 0:1patients are not randomized during the second stage.

At completion of the trial, after the primary outcomes of all ${{{{{\rm{n}}}}}}$ patients become available, we recompute the index of dissimilarity (${W}_{2}$) using all the available data. If ${W}_{2}$ is larger than a predefined threshold ${w}_{2}$, then the EC data are excluded from the final analyses. If ${W}_{2}\le {w}_{2},$ the final trial analyses leverage the EC data.

### Externally controlled trial (ECT) designs

ECTs^5^ (Fig. 1B) are a particular case of the class of designs in Fig. 1, without randomization. The design assumes identical SOC conditional outcome distributions ${\Pr }\left(Y|{{{{{\boldsymbol{X}}}}}},\,A=0,\,S\right)$for the trial and EC populations, which makes the indicator S unnecessary. Patient-level data of the experimental arm and EC data are used to estimate the treatment effect (TE),

1 $${{{{{{\rm{TE}}}}}}}=\mathop{\sum}\limits_{x}\left\{E\left[Y|{{{{{\mathbf{X}}}}}}={{{{{\mathbf{x}}}}}},\,A=1\right]-E\left[Y|{{{{{\boldsymbol{X}}}}}}={{{{{\mathbf{x}}}}}},\,A=0\right]\right\}{{{{{{\rm{Pr}}}}}}}\left({{{{{\mathbf{X}}}}}}={{{{{\boldsymbol{x}}}}}}\right).$$

Here, the expected outcome $E\left[Y|{{{{{\mathbf{X}}}}}}={{{{{\mathbf{x}}}}}},\,A\right]$ of patients receiving experimental (*A = 1*) and control (*A = 0*) treatments with pre-treatment characteristics ${{{{{\mathbf{x}}}}}}$ are weighted by a distribution ${\Pr }({{{{{\mathbf{X}}}}}}={{{{{\mathbf{x}}}}}})$, for example, the distribution of pre-treatment variables ${{{{{\mathbf{X}}}}}}$ in the experimental arm.

We considered different procedures to estimate the TE in (1), including matching^22^, IPW^23^, and MSMs^21^ (see Supplementary Fig. 1). We did not observe substantial differences between these methods and used MSMs in our analyses.

### Testing the null hypothesis of no treatment effects at completion of the study

For ECTs, as well as HTs when ${W}_{2}\le {w}_{2}$, we utilized MSMs^21^ to estimate treatment effects and test the null hypothesis ${H}_{0}:{TE}\le 0$, using the data available at completion of the trial and the EC data. Whereas for RCTs and for HTs with ${W}_{2} \, > \, {w}_{2}$ we utilized only the trial data to estimate treatment effects (estimator: difference of the empirical response rates between the experimental and IC) and test ${H}_{0}$ (test: 2-sample z-test for proportions^31^).

#### Permutation test

We also considered an alternative permutation test (see Supplementary Fig. 7) for HT designs that utilize trial data and EC data (i.e., HTs with ${W}_{2}\le {w}_{2}$). The procedure controls the type I error rate at a predefined ${{{{{\rm{\alpha }}}}}}$-level, both when the standard assumptions of adjustment methods, such as MSM, holds or are violated, for example in settings with unmeasured confounders, or when the conditional outcome distributions ${\Pr }\left(Y|X,\,A=0,\,S\right)$ of the IC ($S=1$) and EC ($S=0$) groups differ. The procedure has three components:

(i) First, a treatment effects estimate $\widehat{{{{{{{\rm{TE}}}}}}}}\left({D}_{{{{{{{\rm{HT}}}}}}}},\;{D}_{{{{{{{\rm{EC}}}}}}}}\right)$is calculated using the HT data and the EC data. Here ${D}_{{{{{{{\rm{HT}}}}}}}}={\{({Y}_{i},\,{X}_{i},\,{A}_{i},\,{S}_{i}=1)\}}_{i\le n}$ indicates the HT data, whereas ${D}_{{{{{{{\rm{EC}}}}}}}}={\{({Y}_{i},\,{X}_{i},\,{A}_{i}=0,\,{S}_{i}=0)\}}_{n \, < \, i\le n+{n}_{{{{{{{\rm{EC}}}}}}}}}$ includes information for *n*_EC_ EC patients. The index *i* identifies the patients.
(ii) Next, we randomly permute ${{{{{\mathscr{l}}}}}}=1,\ldots,\,1000$ times the treatment assignment variables ${\{{A}_{i}\}}_{i\le n}$ in the HT (${A}_{{\rho }_{{{{{{\mathscr{l}}}}}},1},}{A}_{{\rho }_{{{{{{\mathscr{l}}}}}}{,}2}}$… ${A}_{{\rho }_{{{{{{\mathscr{l}}}}}},n}}$), while the assignment variables ${\{{A}_{i}=0\}}_{i \, > \, n}$ in the EC remain identical. For each $1\le {{{{{\mathscr{l}}}}}}{{\le }}1000,$ we obtain a permuted dataset ${D}_{{{{{{{\rm{HT}}}}}}},{\rho }_{{{{{{\mathscr{l}}}}}}}}={\{({Y}_{i},{X}_{i},{A}_{{\rho }_{{{{{{\mathscr{l}}}}}}{{,}}i}},\,{S}_{i}=0)\}}_{i\le n}{and}$compute the estimate ${\widehat{{{{{{\rm{T}}}}}}{{{{{{\rm{E}}}}}}}}_{{{{{{\mathscr{l}}}}}}}}=\widehat{{{{{{{\rm{TE}}}}}}}}({D}_{{{{{{{\rm{HT}}}}}}},{\rho }_{{{{{{\mathscr{l}}}}}}}},\;{D}_{{{{{{{\rm{EC}}}}}}}})$.
(iii) We then estimate the *p*-value (${{{{{{\rm{H}}}}}}}_{0}:{{{{{{\rm{TE}}}}}}}\le 0$) as the proportion of permutations *ℓ* with statistics ${\widehat{{{{{{\rm{T}}}}}}{{{{{{\rm{E}}}}}}}}_{{{{{{\mathscr{l}}}}}}}}$ larger than the actual estimate $\widehat{{{{{{{\rm{TE}}}}}}}}$.

### Evaluation of the trial designs

We evaluated the operating characteristics of the HT, ECT, and RCT designs using model-based simulations and a leave-one-study-out resampling algorithm.

#### Model-based simulations

We generated clinical studies using a parametric model (Table 1) for

- ${\Pr }({{{{{\mathbf{X}}}}}}{|S})$, the distributions of pre-treatment variables in the trial ($S=0$) and EC ($S=1$) populations, and
- ${\Pr }\left(Y|{{{{{\mathbf{X}}}}}},\,A,\,S\right),$ the conditional outcome distributions in the trial ($S=0$) and EC ($S=$1) populations.

We considered scenarios where the distributions of pre-treatment variables (a) and the conditional outcome distributions (b) differ between the two populations (*S* = 0, 1), as well as scenarios with unmeasured confounding.

#### Leave-one-study-out resampling algorithm

**Fig. 3:**
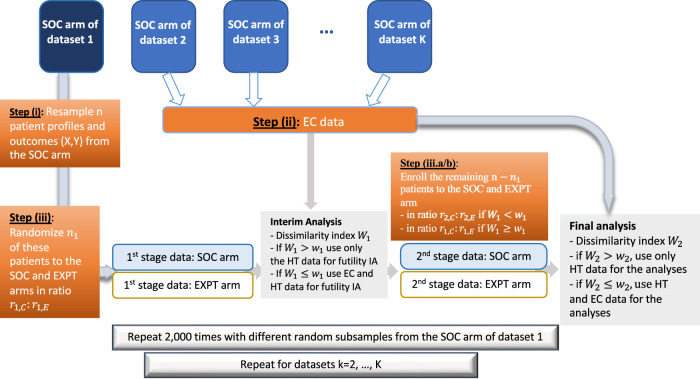
Graphical representation of the leave-one-study-out resampling algorithm. Step (i), we randomly sample with replacement n patient profiles and the corresponding outcomes from the control arm (SOC) of study k. Step (ii), we use the control arms of the remaining studies as externally controlled (EC) data. Step (iii) we randomize *n*_1_ of the patients in Step (i) to the experimental treatment (EXPT) and the SOC arms of our in silico trial and compute the index ${{{{{{\rm{W}}}}}}}_{1}.$ If ${{{{{{\rm{W}}}}}}}_{1}\le {{{{{{\rm{w}}}}}}}_{1}$(${{{{{{\rm{W}}}}}}}_{1} \, > \, {{{{{{\rm{w}}}}}}}_{1}$), the futility interim analysis (IA) leverages (does not leverage) EC data, and we use the ratio r_2,C_:r_2,E_ (r_1,C_:r_1,E_) for the remaining *n*_2_ = *n*−*n*_1_ patients during the 2nd stage. For the final analysis, we recompute the dissimilarity index ${{{{{{\rm{W}}}}}}}_{2}$, and use (don’t use) EC data for inference on treatment effects if ${{{{{{\rm{W}}}}}}}_{2}\le {{{{{{\rm{w}}}}}}}_{2}$(${{{{{{\rm{W}}}}}}}_{2} \, > \, {{{{{{\rm{w}}}}}}}_{2}$). We repeated these Steps (i) to (iii) 2000 times using different random samples.

To evaluate the operating characteristics of the HT design we used a resampling scheme similar to the one described by Ventz et al.^5^ applied to datasets from completed clinical trials and electronic health records in ES-SCLC and GBM (see Fig. 3 and Supplementary Fig. 6). The algorithm provides estimates of the operating characteristics, including type I error rate, power and the average sample size.

#### ES-SCLC datasets

We used patient-level data available at Project Data Sphere^27^ from three randomized Phase III clinical trials: CALGB-9732^11^ (*N* = 283, NCT00003299), Pirker et al.^13^ (*N* = 232, NCT00119613), and GALES^24^ (*N* = 455, NCT00363415). For the Pirker et al. study, a random subsample containing 80% of the original study population was available. The datasets are available for download (via the NCT-id) from Project Data Sphere^27^ at https://data.projectdatasphere.org/. We used data from patients who received etoposide in combination with platinum-based cisplatin (CALGB-9732, Pirker et al., GALES) or carboplatin (Pirker et al.) chemotherapy; both treatments were SOC regimens in ES-SCLC. The statistical procedure to estimate the treatment effects in ECTs and HTs assume identical conditional outcome distributions, given the available pre-treatment characteristics, for these two SOC regimes. The comparison of cisplatin and carboplatin has been previously discussed^32^. Supplementary analysis using data on patients randomized to the control arm of NCT00119613, which received either etoposide plus carboplatin or etoposide plus cisplatin supported this assumption (Log-rank test: p-value 0.4). Nonetheless, undetected differences between these two regimes could impact the operating characteristics of trial designs that leverage EC data.

#### GBM datasets^5,8^

We used patient-level data from a phase III study (Chinot et al.^16^ [NCT00943826], 460 patients), two phase II studies (Cho et al.^33^ [PMID: 22120301], 16 patients; Lee et al.^34^ [NCT00441142], 29 patients) and two real-world datasets^5^ (378 and 305 patients) from DFCI and UCLA. We only used data from patients treated with temozolomide and radiation therapy (TMZ+RT), the SOC in GBM^17^. Pre-treatment variables included age, sex, Karnofsky performance status, MGMT methylation status, and extent of tumor resection^35–37^ (see Supplementary Table 5).

#### Algorithm

For each ES-SCLC (or GBM) study, the algorithm repeatedly samples at random, without replacement, a subset of patients from the control arm. These subsets are used to mimic the data generated during the HTs. Patient-level data from the control arms of the remaining ES-SCLC (or GBM) datasets are used as EC.

Specifically, for each ES-SCLC (or GBM) study *k*, we randomly generated 2000 trials by repeating the following steps (see also Fig. 3) 2000 times (using different computer-generated random subsamples):

(i) Randomly subsample (with replacement) n patient profiles **X** and the corresponding outcomes Y from the control arm (SOC) of study *k*.
(ii) Use the control arms of the remaining studies as EC data.
(iii) Randomize (with replacement) ${n}_{1}$ of the patients in Step (i) to the experimental and control arms of the in silico HT in ratio ${r}_{1,C}:{r}_{1,E}$ and compute the index ${W}_{1}.$
  (iii.a) If ${W}_{1}\le {w}_{1}$, use the ratio r_2,C_:r_2,E_ for the remaining ${n}_{2}{=n-n}_{1}$ patients in stage 2.
  (iii.b) If ${W}_{1} \, > \, {w}_{1}$, use the ratio r_1,C_:r_1,E_ for the remaining ${n}_{2}{=n-n}_{1}$ patients in stage 2.
(iv) Use the output of Steps (i-iii) to generate an in silico HT trial, including the futility IA and, if the in silico HT is not discontinued, final hypothesis testing (Fig. 1A).

We used the statistical software R^38^ to implement the algorithm.

The ${n}_{1}$ patients (randomly selected) from the control arm of study *k* in Step (iii.a) allowed us to mimic the data of the experimental and IC arms of the HT during the first stage of the study, whereas the remaining ${n}_{2}$ patients in Step (iii.b) mimicked the second stage of the HT. In these in silico HTs, the treatment effect is null by construction of the algorithm because the outcome distributions in the two arms of the trial are identical.

To evaluate the power of the HT design, we added a component to Step (iii) of the algorithm (see Supplementary Fig. 6), which allowed us to produce in silico HTs with positive treatment effects. For each enrollment i to the experimental arm (${A}_{i}=1$), if the patient had a negative response (${Y}_{i}=0$), we randomly generate a binary random variable ${R}_{i},\,{{{{{\rm{with}}}}}}\,{{{{{{\rm{Pr}}}}}}}\left({R}_{i}=1\right)=\pi$, representative of the treatment effect for patient i. If ${R}_{i}=1$, then the negative outcome is relabeled as a positive outcome (i.e., we set ${Y}_{i}=1$). If ${R}_{i}=0$, then the outcome remains unchanged (${Y}_{i}=0$). We used $\pi=0.4$ for ES-SCLC and $\pi=0.5$ for GBM analyses reported in the “Results”, and different values of ${{{{{\rm{\pi }}}}}}$ for analyses reported in the Supplementary Information.

### Reporting summary

Further information on research design is available in the Nature Research Reporting Summary linked to this article.

## Supplementary information

Supplementary Information

Description of Additional Supplementary Files

Supplementary Software 1

Reporting Summary

## Data Availability

Source data are provided with this paper. The SCLC datasets (NCT00003299, NCT00119613, NCT00363415, NCT01439568, NCT00453154) used in this study are available for download from Project Data Sphere^27^ at https://data.projectdatasphere.org/. The GBM data were not generated for the purpose of this study, are protected and are not publicly available due to data privacy laws. We received permission to use the GBM data from Brian M. Alexander, Patrick Y. Wen and Rifaquat Rahman, and since restrictions apply to the availability of these data, please contact Drs Alexander (Brian_Alexander@dfci.harvard.edu), Wen (Patrick_Wen@dfci.harvard.edu) and Rahman (RRAHMAN@BWH.HARVARD.EDU) for access to these data. De-identified patient-level data (treatment outcomes and pre-treatment patient characteristics) will be shared upon request starting 1 months after publication for up to 3 years for research purposes. The remaining data are available within the Article, Supplementary Information or Source Data file. Source data are provided with this paper.

## Source data

Source Data

## Code availability

R code used to generate the HTs, ECTs and RCTs, and implement the leave-one-study-out resampling algorithm are available as ‘Supplementary Software 1’.
