# Supplementary material for: The design and evaluation of hybrid controlled trials that leverage external data and randomization
Source: Nat Commun. 2022 Oct 2;13:5783. doi: 10.1038/s41467-022-33192-1 (PMC9527257; doi:10.1038/s41467-022-33192-1)
Supplement: Supplementary file 1 — Supplementary Information [file 41467_2022_33192_MOESM1_ESM.pdf]

Supplementary Information for "The Design and Evaluation of Hybrid Controlled Trials that Leverage External Data and Randomization"

Steffen Ventz[1], Sean Khozin[2], Bill Louv[3], Jacob Sands[4], Patrick Y. Wen[5], Rifaquat Rahman[6], Leah Comment[7], Brian M. Alexander[6,7]* and Lorenzo Trippa[8,9,]*

1 Division of Biostatistics, University of Minnesota, Minneapolis, MN
2 Janssen R&D, Johnson & Johnson, New Brunswick NJ
3 Project Data Sphere, Morrisville, NC
4 Department of Medical Oncology, Dana-Farber Cancer Institute, Boston, MA
5 Center for Neuro-Oncology, Dana-Farber Cancer Institute, Boston, MA
6 Department of Radiation Oncology, Dana-Farber Cancer Institute, Boston, MA
7 Foundation Medicine, Inc., Cambridge, MA
8 Department of Data Science, Dana-Farber Cancer Institute, Boston, MA
9 Department of Biostatistics, Harvard School of Public Health, Boston, MA

*These authors contributed equally

Correspondence:
Steffen Ventz, PhD
Division of Biostatistics
University of Minnesota
A460 Mayo Building, MMC 303
420 Delaware Street SE
Minneapolis, MN 55455
Office phone: (617) 259-8412
steffen.ventz.81@gmail.com

Supplementary Methods

Dissimilarity measure

The HT design (see Methods) utilizes dissimilarity statistics W_ℓ at completion of the 1st phase ($\ell = 1$) of the study and at completion of the trial ($\ell = 2$). The statistics W_ℓ quantifies evidence against the hypothesis of identical conditional binary outcome distributions $\Pr(Y_i = 1|X_i, A_i = 0, S_i)$ for the IC ($A_i = 0, S_i = 0$) and EC ($A_i = 0, S_i = 1$) populations based on the available data at the interim ($\ell = 1$) and final ($\ell = 2$) analyses.

For both analyses (ℓ =1,2), we estimate the parameters (maximum likelihood estimation) of two nested binary regression models M_1 and M_2,

$$M_1\colon\ Pr_1(Y_i = 1|X_i = x, A_i = 0, S_i) = F\left(\beta_0 + \sum \beta_j\, x_j\right),$$

where $F(t) = 1/(1 + \exp(-t))$, and

$$M_2\colon\ \Pr_2(Y_i = 1|X_i = x, A_i = 0, S_i) = F\left(\beta_0 + \gamma_0 S_i + \sum \beta_j\, x_j + \sum \gamma_j * x_j * S_i\right).$$

Under M_1, patients in the IC and EC population have identical conditional outcome distributions. Whereas under model M_2 the IC and EC groups can have different conditional outcome distributions, i.e. $\Pr_2(Y_i|X_i, A_i = 0, S_i)$ for $S_i = 0{,}1$ can be different.

Model M_1 is nested within model M_2 (i.e., M_1 corresponds to M_2 when $\gamma_j = 0$ for all $j = 0, 1, \dots, J-1$, interaction parameters), where J indicates the difference between the number of parameters in model M_2 and M_1.

We compute the likelihood ratio statistics[1]

$$W_\ell = 2 * \left\{ \log Pr_2(Data;\ \hat{\beta}^{M_2},\ \hat{\gamma}^{M_2})) - \log Pr_1(Data; \hat{\beta}^{M_1})\right\},$$

where $Data = \{(Y_i, X_i, A_i, S_i)\}_i$ indicates the available (internal and external) data at the time of (interim or final) analysis. Here $\hat{\beta}^{M_1}$ and $(\hat{\beta}^{M_2},\ \hat{\gamma}^{M_2})$ are the maximum likelihood estimates for the parameters in model M_1 and M_2 .

Under mild assumptions[1], the statistics W_ℓ has approximately a central χ^2_J-distribution with J degrees of freedom[1] and expected value $E_{M_1}[W_\ell] = J$ when Model 1 holds. While, if Model 2 holds, then W_ℓ has approximately a non-central χ^2_J-distribution[2] with J degrees of freedom, non-centrality parameter $\Delta \geq 0$, and $E_{M_2}[W_\ell] = J+\Delta$. The parameter $\Delta \geq 0$ increases with the magnitude of the components of γ and the sample sizes of the EC and IC datasets[1,2]. In summary, both Δ and the statistics W_ℓ tend to increase with the magnitude of γ and the sample size. We use the statistics W_ℓ to quantify the dissimilarity of the conditional outcome distributions.

Futility interim stopping rules

The ECT, HT and RCT designs (Methods) allow for early termination of the study due to insufficient evidence of positive treatment effects (TEs) of the experimental treatment compared to the SOC treatment, $TE = E_{X,S=0}[P(Y = 1|X, A = 1) - E_{X,S=0}[P(Y = 1|X, A = 0)$, (i.e., early futility stopping). Based on the available data at the end of the 1st stage, we compute a one-sided $(1 - \alpha_{IA})$-confidence interval, $(-\infty, U_{\alpha_{IA}})$, for the treatment effect TE (we used $\alpha_{IA} = 0.1$ in our simulations). For HTs when $W_1 > w_1$, and for RCTs, we use standard confidence intervals[3] based on normal approximations for the differences of the response rates of the experimental and IC arms – without using EC data. Whereas for both, the HT design when $W_1 \leq w_1$ and the ECT design, we used confidence intervals based on normal approximations for the estimated treatment effects based on marginal structural models[4](MSMs). If the confidence interval $(-\infty, U_{\alpha_{IA}})$ does not include a minimum targeted treatment effect TE_{min} (we use 0.1 in our analyses), then the study is closed early for futility.

Adjusted treatment effects' estimates

We used MSMs[4] to estimate

$$TE = E_{\Pr(X)}[\, E(Y|X, A = 1) - E(Y|X, A = 0)]$$
$$= E_{Pr(X)}\big[Y|A = 1\big] - E_{\Pr(X)}[Y|A = 0\big], \qquad (S1)$$

in ECTs, where $Pr(X)$ is a reference distribution of pre-treatment patient characteristics. The estimation of the TE with MSMs requires the following main steps:

1) MSMs require the specification of patient-specific weights r_i, for each patient i in the experimental ($A_i = 1$) and in the EC ($A_i = 0$) groups. The weights r_i are functions of the propensity score (PS) estimates $\hat{e}_i$. The PS e_i corresponds to the conditional probability $e_i = Pr(A_i = 1|X_i)$. The estimates $\hat{e}_i$ are computed fitting a logistic model $e_i = (1 + e^{-X_i\eta})^{-1}$ with parameters η.

   Different types of weights r_i can be specified. The definition of r_i depends on the reference distributions $Pr(X)$ in equation (S1). For example, if $Pr(X)$ is the distribution of pre-treatment variables X in the experimental arm (A_i=1), then r_i= $A_i + (1 - A_i)\hat{e}_i/(1 - \hat{e}_i)$.

2) Next, we use a marginal logistic regression model, $E_{Pr(X)}[Y|A; v] = 1/(1 + e^{-v_0 - v_A A})$, to estimates the conditional expectations in (S1). The parameters $v = v(Pr(X)) = (v_0, v_A)$

depend on the reference distribution $Pr(X)$, and are estimated by maximizing the weighted log-likelihood function

$$l(v) = \sum_i r_i \ln \Pr(Y_i|A_i; v) \ (S2).$$

Here the weights $\{r_i\}_i$ combine the patient-specific log-likelihoods $\ln \Pr(Y_i|A_i; v)$, to estimate v in the reference population $Pr(X)$.

Alternatively, one can fit a logistic model $E_{Pr(X)}[Y|A, X^*; v] = 1/(1 + e^{-v_0 - v_A A - v_X X^*})$, given a subset X^* of pre-treatment variables, via weighted maximum likelihood estimation (as in S2). When $Pr(X)$ in (S1) corresponds to the distribution of pre-treatment variables in the (combined) EC and ECT study population and $r_i = \frac{A_i}{\hat{e}_i} + \frac{1-A_i}{1-\hat{e}_i}$, we can use G-computations[5,6], and obtain the estimate

$$\widehat{TE}_G = (N + N_{EC})^{-1} \sum_i \{ E_{Pr(X)}[Y|A = 1, X_i^*; v] - E_{Pr(X)}[Y|A = 0, X_i^*; v]\}.$$

Censoring and data imputation for binary primary outcomes

In our resample-based evaluation of the ECT and HT designs with binary outcomes (overall survival [OS] after t_E months), the OS times ($Y_{OS,i}$) of some patients in the ES-SLCL datasets were censored at time $t_i < t_E$, within the first t_E months since randomization, and the binary outcome $Y_i = 1(Y_{OS,i} > t_E)$ was therefore unknown. For these patients we used data imputation[7,8] based on estimates of the conditional probability $Pr(Y_{OS,i} \geq t_E | Y_{OS,i} > t_i, X_i)$ using study-specific proportional hazards models.

Power of the ECT and RCT designs

For any testing procedure (e.g., t-test), the power increases with (i) the treatment effect and (ii) the arm-specific sample sizes. For a fixed treatment effect, the power of the test increases with the number of patients in the control and in the experimental arms. Intuitively, a large sample size corresponds to low variability of the treatment effect estimate, and in turn to accurate discrimination between superior and non-superior experimental treatments.

Hypothesis testing with a RCTs that enrolls n patients typically involves only the outcome data. In contrast, the ECT design augments the internal sample size (n patients, all assigned to the experimental arm) of the study with an additional EC dataset of n_{EC} patients treated with the SOC. Hypothesis testing with the ECT design involves data from $n_{EC} + n$ patients. This augmented size

explains differences in the power between the RCT and ECT designs.

We provide a stylized example for a clinical trial with n=50 patients. For simplicity we assume that there are no relevant pre-treatment variables to adjust for (i.e., there are no confounders, this assumption could be easily relaxed). We compare:

(a) the RCT design, with 1:1 randomization. The RCT enrolls 25 patients for the experimental arm and 25 patients for the IC arm.

(b) The ECT design, with an additional EC dataset with $n_{EC} = 500$ patients. The ECT assigns to all 50 patients the experimental treatment and leverages the 500 EC datapoints.

By using 500 additional data points (50 vs 550), the ECT design has higher power than the RCT design. For example, for binary outcomes and response probabilities $(p_0, p_1) = (0.6, 0.85)$ for the control and experimental treatments, the RCT with a z-test for proportions has a 64% power, while the ECT with a z-test has >90% power.

Hybrid design **for time-to-event endpoints**

The HT design for time-to-event endpoints (e.g. OS, PFS, etc.) is nearly identical to the one for binary outcomes. At completion of the first stage, after enrollment of the first n_1 patients in ratio $r_{1,C}$:$r_{1,E}$, an IA is conducted to determine if the trial should be terminated early for futility, and potentially to update the randomization ratio to $r_{2,C}$:$r_{2,E}$ for the remaining n_2 patients during the second stage. These two decisions are supported by an index of dissimilarity.

We use the log partial-likelihood ratio statistics[9] between two nested Cox proportional hazards models $W_1 = 2 * \{\, l_2(\hat{\beta}^{M_2}, \hat{\gamma}^{M_2})) - \, l_1(\hat{\beta}^{M_1})\}$. Here $l_j, j = 1,2$, indicates the log partial-likelihood of two nested Cox models. The approach is nearly identical to the binary case (Section 1.1). The first model M_1 - with patient-specific hazard rate proportional to $\exp(\sum \beta_j\, x_j)$ - assumes that the conditional distribution $P_1(Y_i \geq t | X_i = x, A_i = 0, S_i)$ does not dependent on S_i. Whereas model M_2, with hazard rate proportional to $\exp(\gamma_0 S_i + \sum \beta_j\, x_j + \sum \gamma_j * x_j * S_i)$, allows for differences $P_2(Y_i \geq t | X_i = x, A_i = 0, S_i = 0) \neq P_2(Y_i \geq t | X_i = x, A_i = 0, S_i = 1)$ between the conditional survival distributions $S_i = 0{,}1$.

At completion of the trial, after $N \leq n$ events became available, we re-compute the index of dissimilarity (W_2) using all the available data. If W_2 is larger than a pre-defined threshold w_2, then the EC data are excluded from the final analyses. If $W_2 \leq w_2$, then the final trial analyses leverage the EC data.

For HTs with $W_2 \leq w_2$, we utilized a Cox proportional hazards MSM[4] to estimate the hazard ratio (HR) between the experimental and SOC treatments and to test the null hypothesis $H_0: HR \geq 1$, using the data available at completion of the trial and the EC data. For HTs with $W_2 > w_2$ we utilize only the trial data to estimate the HR (Cox model) between the experimental and IC groups, and test H_0 (test: log-rank test[9]).

Study<br>NCT number	CALGB-9732[11]<br>0000-3299	GALES[12]<br>0036-3415	Pirker et al.[13]<br>0011-9613	Salgia et al.[14]<br>0143-9568	CALGB-30504[15]<br>0045-3154
Age	Yes	Yes	Yes	Yes	Yes (<65, ≥65)
Sex	Yes	Yes	Yes	Yes	Yes
ECOG PS	Yes	Yes	Yes	**No**	Yes
Smoking before the study	**No**	**No**	**No**	Yes	**No**
Weight loss within last 6 month	**No**	**No**	**No**	**No**	**No**
Hemoglobin level at baseline	**No**	Yes	Yes	Yes	**No**
Baseline lactate dehydrogenase in normal range	**No**	Yes	Yes	**No**	**No**
HER2	**No**	**No**	**No**	**No**	**No**
CYFRA 21-1	**No**	**No**	**No**	**No**	**No**
TP53	**No**	**No**	**No**	**No**	**No**

Supplementary Table 1: Availability of potential confounding pre-treatment variables in the ES-SCLC datasets.

Study<br>NCT number	CALGB-9732[11]<br>0000-3299	GALES[12]<br>0036-3415	Pirker et al.[13]<br>0011-9613	Salgia et al.[14]<br>0143-9568	CALGB-30504[15]<br>0045-3154
**Enrollments**	283	455	232	42	41
**Sex**					
Male	130 (46)	330 (73)	159 (69)	17 (40)	20 (49)
Female	153 (54)	125 (27)	73 (31)	25 (60)	21 (51)
**Age**					
<65	96 (34)	180 (40)	80 (34)	26 (62)	16 (39)
≥65	187 (66)	275 (60)	152 (66)	16 (38)	25 (61)
**Race**					
White	255 (90)	379 (83)	232 (100)	39 (93)	40 (98)
African Americans	17 (6)	8 (2)	0 (0)	2 (5)	1 (2)
Native Americans	2 (1)	0 (0)	0 (0)	0 (0)	0 (0)
Asian	1 (0)	61 (13)	0 (0)	0 (0)	0 (0)
Other	8 (3)	7 (2)	0 (0)	1 (2)	0 (0)
**ECOG PS**					
0	80 (28)	121 (27)	0 (0)	0 (0)	17 (41)
1	189 (67)	277 (61)	181 (78)	0 (0)	15 (37)
2	13 (5)	55 (12)	51 (22)	0 (0)	9 (22)
Missing	*1 (0)*	*2 (0)*	*0 (0)*	*42 (100)*	*0 (0)*

Supplementary Table 2: Distribution of pre-treatment variables for the *control* arms of the five ES-SCLC studies. For each study and pre-treatment variable, the table reports the number (proportion) of patient per category. Source data are provided as a Source Data file.

Model<br>Variables	Stratified Cox model<br>HR (95%-CI)	 2-sided p-value	Random effects Cox model<br>HR (95%-CI)	 2-sided p-value
**Sex (ref. Female)**				
Male	1.45 (1.23, 1.72)	<0.001	1.42 (1.21, 1.68)	<0.001
**Age (ref.>=65)**				
	0.70 (0.68, 0.82)	<0.001	0.71 (0.60, 0.83)	<0.001
**ECOG-PS (ref. 0)**				
1	1.28 (1.00, 1.59)	0.0024	1.28 (1.03, 1.59)	0.02
2	2.54 (2.24, 3.44)	<0.001	2.43 (1.81, 3.25)	<0.001
**Race(ref. white)**				
Black	1.30 (0.83, 2.02)	0.244	1.34 (0.86, 2.08)	0.187
Indian/Alaskan	0.40 (0.09, 1.66)	0.197	0.42 (0.10, 1.76)	0.227
Asian	0.94 (0.58, 1.51)	0.781	0.87 (0.55, 1.40)	0.568
Other	1.16 (0.66, 2.05)	0.599	1.18 (0.67, 2.07)	0.565
**Random effects**				
CALGB-9732[11]	-	-	1.08	-
GALES[12]	-	-	0.91	-
Pirker et al.[13]	-	-	1.03	-

Supplementary Table 3: Pre-treatment patient characteristics associated with overall survival (OS) for ES-SCLC patients treated with etoposide in combination with platinum-based chemotherapy (EP). Estimated hazard ratios (HRs) in a multivariable Cox regression model with baseline hazard rate stratified by study (2nd column) and a non-stratified multivariable Cox regression model with study specific random intercepts (3rd column). Source data are provided as a Source Data file.

Model<br>Variables	Random effects Logistic Regression<br>OR (95%-CI)	 p-value
**Sex (ref. Female)**		
Male	1.42 (1.21, 1.68)	<0.001
**Age (ref.>=65)**		
	0.71 (0.60, 0.83)	<0.001
**ECOG-PS (ref. 0)**		
1	1.28 (1.03, 1.59)	0.02
2	2.43 (1.81, 3.25)	<0.001
**Race (ref.)**		
Black	1.34 (0.86, 2.08)	0.187
Indian/Alaskan	0.42 (0.10, 1.76)	0.227
Asian	0.87 (0.55, 1.40)	0.568
Other	1.18 (0.67, 2.07)	0.565
**Random effects**		
CALGB-9732[11]	1.08	-
GALES[12]	0.91	-
Pirker et al.[13]	1.03	-

Supplementary Table 4: Logistic regression with study-specific random intercepts. Estimated odds ratios (ORs) for the effect of pre-treatment patient characteristics on overall survival after 9 months of EP treatment. Source data are provided as a Source Data file.

**Study**<br>**PubMed ID**	DFCI[16]	UCLA[16]	Chinot et al[17]<br>PM24552318	Lee at al.[18]<br>PM25910950	Cho et al.[19]<br>PM22120301
**Sample size**<br>**OS Events**	378<br>269	305<br>265	460<br>344	29<br>24	16<br>15
**Age**					
Median	58	57	57	58	59
Range	18-91	20-84	18-79	26-73	36-69
SD	13	13	10	11	11
**Sex (%)**					
Females	0.43	0.36	0.36	0.45	0.5
Males	0.57	0.64	0.64	0.55	0.5
**KPS (%)**					
<=80	0.55	0.39	0.31	0.24	0.44
>80	0.45	0.61	0.69	0.76	0.56
**Resection (%)**					
Biopsy	0.14	0.22	0.09	0.21	0
Sub Total	0.47	0.47	0.49	0.48	0.31
Gross Total	0.39	0.31	0.42	0.31	0.69
**MGMT (%)**					
Unmethylated	0.43	0.71	0.67	0.86	0.43
Methylated	0.57	0.29	0.32	0.14	0.56

Supplementary Table 5: Distribution of pre-treatment patient characteristics for the temozolomide plus radiation (TMZ+RT) groups in the collection of GBM datasets. Source data are provided as a Source Data file. *Abbreviations*: KPS, Karnofsky performance status; MGMT, O6-methylguanine-DNA methyltransferase, SD, standard derivation; OS, overall survival.

Model	Stratified Cox model		Random effects Cox model	
Variables	HR (95%-CI)	2-sided p-value	HR (95%-CI)	2-sided p-value
**Sex (ref. Female)**				
Male	1.14 (0.96, 1.38)	0.133	1.15 (0.96, 1.38)	0.119
**Age**				
linear	1.04 (1.03, 1.05)	$2.56*10^{-16}$	1.04 (1.03, 1.05)	$1.24*10^{-16}$
**KPS (ref. <90)**				
>= 90	0.75 (0.62, 0.91)	0.003	0.76 (0.63,0.91)	0.003
**MGMT (ref. unmethylated)**				
Methylated	0.44 (0.36,0.53)	$1.80*10^{-17}$	0.44 (0.36, 0.53)	$4.38*10^{-16}$
**Resection (ref. biopsy)**				
Sub total	1.32 (0.91, 1.93)	0.139	1.29 (0.89, 1.87)	0.178
Gross total	1.28 (1.06, 1.53)	0.008	1.27 (1.06, 1.52)	0.009
**Random effects**				
DFCI[16]	-	-	0.9998	-
UCLA[16]	-	-	0.9998	-
Chinot et. al[17]	-	-	1.0001	-
Cho et al.[18]	-	-	1.0001	-
Lee et al.[19]	-	-	1.0000	-
SD of random effects	-	-	0.004	-

Supplementary Table 6: Pre-treatment patient characteristics associated with overall survival for GBM patients treated with TMZ+RT. Estimated hazard-ratios in a multivariable Cox regression model with baseline hazard rate stratified by study (2nd column) and a non-stratified multivariable Cox regression model with study specific random intercepts (3th column). Source data are provided as a Source Data file. *Abbreviations*: KPS, Karnofsky performance status; MGMT, O6-methylguanine-DNA methyltransferase, SD; standard derivation; HR, hazard ratio.

Design	$r_{2,C}:r_{2,E}$	HT									ECT			RCT		
		1:1			1:2			0:1			0:1			1:1		
	π	Power	% of trials stopped at IA	Average sample size	Power	% of trials stopped at IA	Average sample size	Power	% of trials stopped at IA	Average sample size	Power	% of trials stopped at IA	Average sample size	Power	% of trials stopped at IA	Average sample size
DFCI[16]	0.4	61	2	99	69	1	99	74	2	99	79	1	100	46	0	100
	0.5	77	0	100	82	0	100	88	0	100	92	0	100	63	0	100
	0.6	90	0	100	89	0	100	94	0	100	99	0	100	78	0	100
	0.7	95	0	100	95	0	100	97	0	100	100	0	100	92	0	100
UCLA[16]	0.4	58	2	99	64	2	99	70	2	99	62	0	100	41	1	100
	0.5	73	1	100	78	1	100	85	1	100	86	0	100	58	0	100
	0.6	86	0	100	88	0	100	92	0	100	96	0	100	75	0	100
	0.7	91	0	100	93	0	100	95	0	100	99	0	100	87	0	100
Chinot et. al[17]	0.4	57	2	99	60	2	99	69	2	99	67	1	100	42	1	100
	0.5	73	0	100	78	1	100	84	1	100	85	0	100	58	0	100
	0.6	86	0	100	88	0	100	92	0	100	96	0	100	74	0	100
	0.7	92	0	100	94	0	100	95	0	100	100	0	100	87	0	100

Supplementary Table 7: Resampling-based evaluation of the operating characteristics of the hybrid trials (HTs), externally controlled trials (ECTs) and randomized clinical trials (RCTs) designs in GBM. We used individual-level data from patients treated with TMZ+RT from five GBM data sets. Rows 4-7, 8-11 and 12-15 show results for an experimental treatment with a positive treatment effect, when $\pi = 0.4, 0.5, 0.6$ and 0.7. Here π indicates the conditional probability of a positive response to the experimental treatment given a counterfactual negative response to the control treatment. We report the power, the proportion of trials stopped early for futility, and the average sample size across 2,000 *in silico* trials. Source data are provided as a Source Data file.

Scenarios	Distribution of pre-treatment variables in the EC population			Effect of pre-treatment variables on the outcome in the EC (and HT) population			Response rates for the EC, IC and EXPT		
	p_1	p_2	p_3	$\theta_{S,1}$	$\theta_{S,2}$	$\theta_{S,3}$	EC	IC	EXPT
1	0.2	0.8	0.5	0.5	-0.5	0.0	0.43	0.50	0.40
2	0.2	0.8	0.1	0.5	-0.5	1.5	0.46	0.66	0.58
3	0.2	0.8	0.9	0.5	-0.5	1.5	0.73	0.66	0.58
4	0.2	0.8	0.1	0.5	-1.5(1.5)	1.5	0.30	0.66	0.58
5	0.2	0.8	0.9	0.5	-1.5(1.5)	1.5	0.55	0.66	0.58

Supplementary Table 8: Inferiority scenarios (ET<0). We consider three binary pre-treatment variables $X = (X_1, X_2, X_3)$. The variable X_3 is not available and is not used in the interim and final analyses. For patients enrolled in the hybrid trial (HT), the three pre-treatment variables are independent, with $p(X_j = 1) = 0.5$ for $j = 1,2,3$. Columns 2-4 report the distribution $p(X_j = 1)$ of the three independent variables in the external control (EC) population. Patient outcomes Y, given the pre-treatment variables, were randomly generated from a logistic model, $p(Y = 1|X, A, S) = F(\delta A + X'\theta_S), A = 0,1$ and $S = 0,1$, where $F(t) = 1/(1 + \exp\{-t\})$. Columns 5-7 show the effects ($\theta_{S,j}$, log odds-ratio) of the pre-treatment variables X_j on the expected outcome Y in the EC (S=1) and HT (S=0) populations. When $\theta_{0,j} = \theta_{1,j}$ we omit the value in parenthesis ($\theta_{0,j}$). The treatment effect (TE, log odds ratio) for inferior experimental treatments equals $\delta = -0.4$. Columns 8-10 show the average response probability for the EC ($A = 0, S = 1$), the internal control (IC) ($A = 0, S = 0$), and the experimental treatment (EXPT, $A = 1, S = 0$) populations with and without treatment effects.

**Design**<br>assignment ratio $r_{2,C}:r_{2,E}$	**HT**<br>**1:1**	**HT**<br>**1:2**	**HT**<br>**0:1**	**ECT**<br>**0:1**	**RCT**<br>**1:1**
	Scenario 1 (no unmeasured confounding)				
Type I error rate (%)	1	0	1	0	0
% of trials stopped at IA	49	38	37	90	25
Average study duration	18	19	19	13	21
Average sample size	91	97	98	66	105
	Scenario 2 (unmeasured confounding)				
Type I error rate (%)	1	1	1	1	1
% of trials stopped at IA	25	22	23	90	22
Average study duration	21	21	21	13	21
Average sample size	105	107	106	66	107
	Scenario 3 (unmeasured confounding)				
Type I error rate (%)	1	1	1	20	1
% of trials stopped at IA	29	25	25	21	21
Average study duration	20	21	21	21	21
Average sample size	103	105	105	107	107
	Scenario 4 (unmeasured confounding)				
Type I error rate (%)	1	1	1	0	1
% of trials stopped at IA	22	22	22	100	21
Average study duration	21	21	21	12	21
Average sample size	107	107	107	60	107
	Scenario 5 (unmeasured confounding)				
Type I error rate (%)	1	1	1	95	1
% of trials stopped at IA	44	33	33	1	22
Average study duration	19	20	20	24	21
Average sample size	94	100	100	119	107

Supplementary Table 9: Operating characteristics of the hybrid trials (HTs), externally controlled trials (ECTs) and randomized clinical trials (RCTs) designs when the ET<0, for different distributions of measured (X_1, X_2) and unmeasured (X_3) prognostic patient pre-treatment characteristics (see Table S9 for details). For each scenario we report the type I error rate, the proportion of generated trials stopped early at IAs for futility and the average sample size/study duration across 2,000 simulations. Source data are provided as a Source Data file.

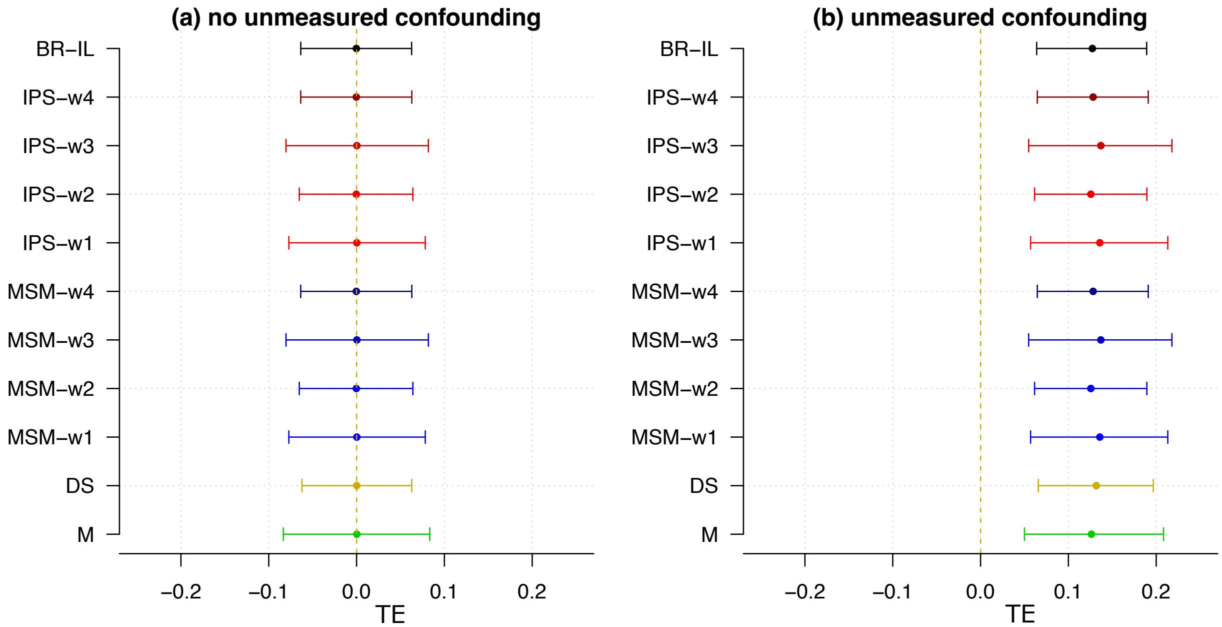

Supplementary Figure 1: Comparison of different methods to estimate covariate adjusted treatment effect estimates. Panel A reports treatment effect estimates (dot=average value, bars=10th and 90th percentile) across 10,000 generated ECT trials (n=75) according to scenario 1 in Table 1, without unmeasured confounding, when the true treatment effect is null (TE=0, vertical brown dashed line). We consider matching (M), direct standardization (DS), marginal structural models (MSMs), inverse-probability weighting (IPW), and binary-regression with identity-link (BR-IL) function. For IPW and MSM we use different reference distributions $w_r, r = 1, \dots, 4$ (see (1) in the main document and Ventz et al.[10]), which correspond to the empirical distribution of X in the experimental arm (r=1), in the EC population (r=2), in the HT and EC combined groups (r=3), and in the overlap of the two populations (r=4). Panel B shows the same operating characteristics when there is unmeasured confounding between the study and EC population (according to scenario 2 in Table 1) and the true treatment effect is null (TE=0). Source data are provided as a Source Data file.

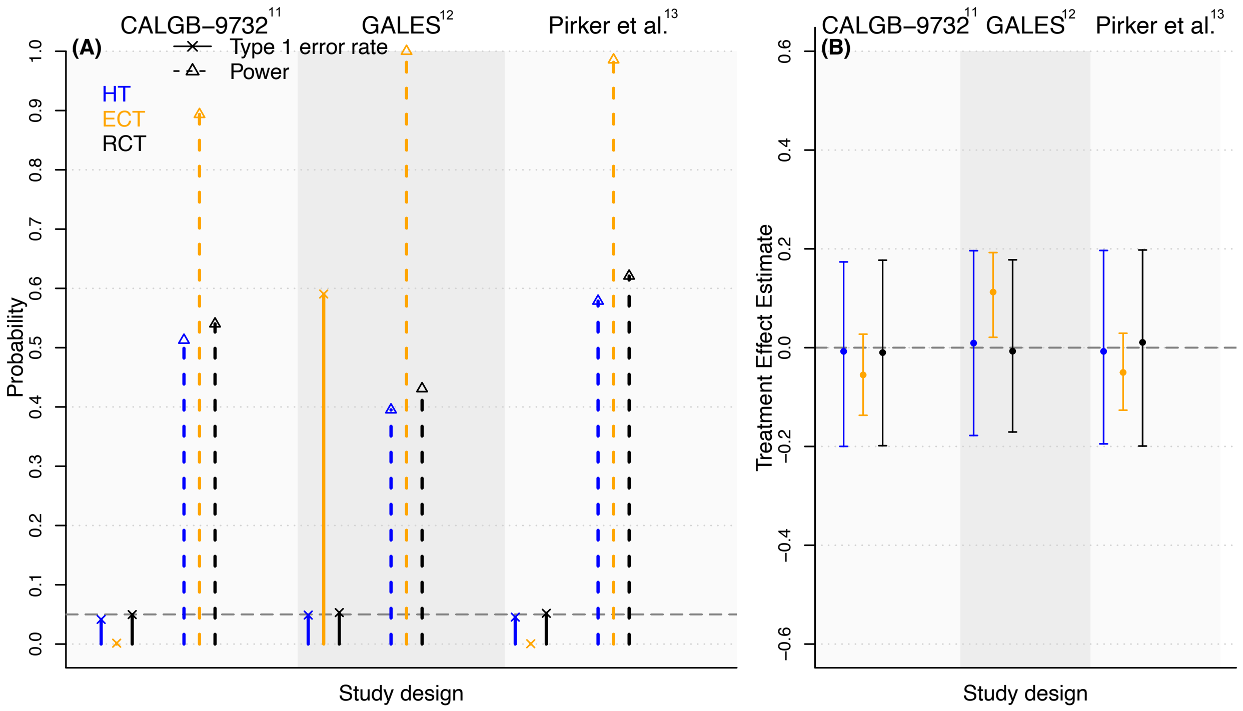

Supplementary Figure 2: Operating characteristics of *in silico* hybrid trials (HTs), externally controlled trials (ECTs) and randomized clinical trials (RCTs, n=75 for all trial designs) generated by resampling the control arms of the ES-SCLC studies. For HTs we used final analyses based on a permutation test (see Methods). Panel A shows type I error rates (solid vertical lines with a cross) and power (dotted vertical lines with an arrow) across 2,000 *in silico* trials (n=75). Panel B illustrates the variability/bias of the treatment effect estimates; the dots indicate the average treatment effect estimates across 2,000 *in silico* trials (n=75) and the vertical bars indicate the 5% and 95% percentiles across these *in silico* trials using the leave-one-study-out resampling algorithm (TE=0). Source data are provided as a Source Data file.

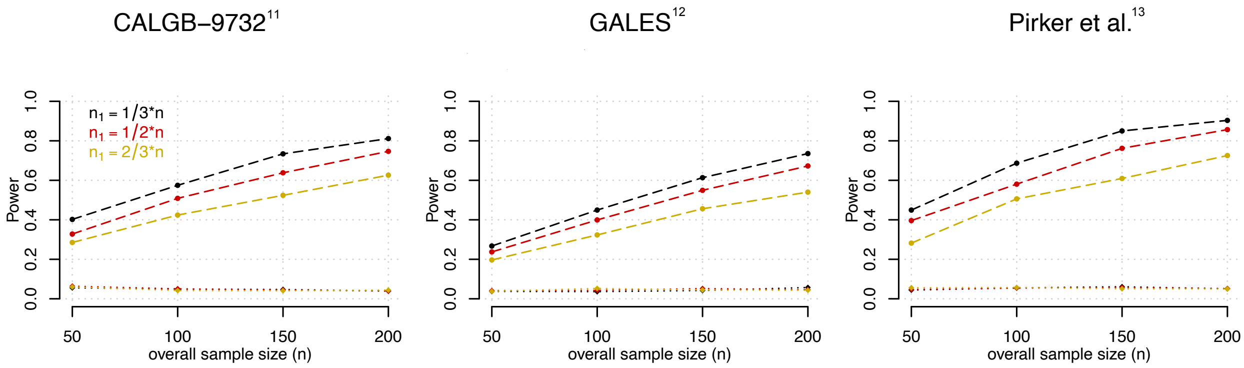

Supplementary Figure 3: Power (dashed curve) and type I error rates of the HT design (final analyses based on a permutation test, $r_{1,C}: r_{1,E} = 1:1$ and $r_{2,C}: r_{2,E} = 0:1$) across different sample size n (x-axis). We used the leave-one-study-out resampling algorithm. Different colors correspond to different 1st stage sample sizes n_1, $\frac{n_1}{n} = \frac{1}{3}, \frac{1}{2}, \frac{2}{3}$. Source data are provided as a Source Data file.

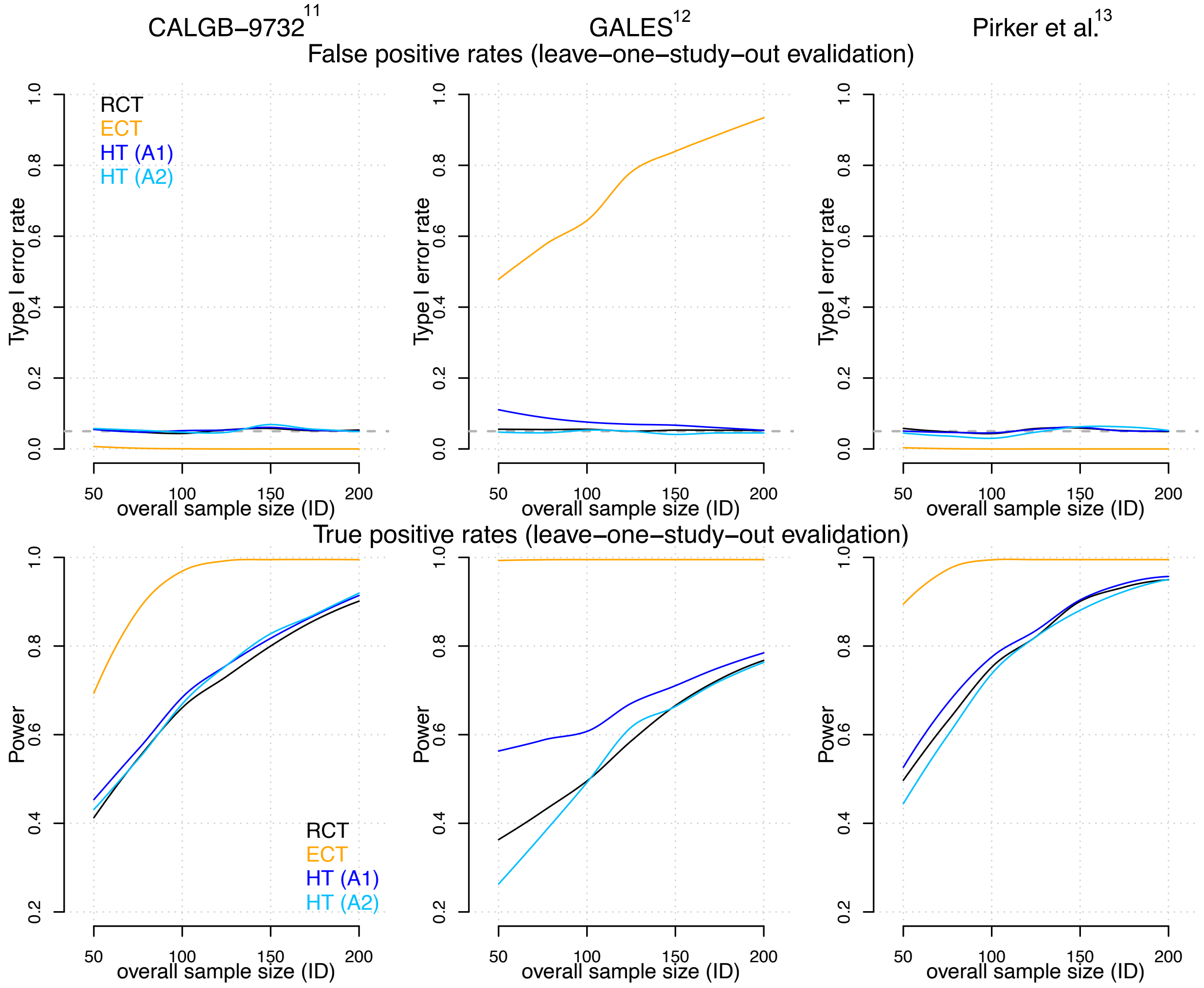

Supplementary Figure 4: Type I error rates (1st row) and power (2nd row, $\pi = 0.4$, in the leave-one-study-out resampling algorithm) of different trial designs across a range of overall sample sizes n=50, …, 200. We consider the hybrid trial (HT, with $r_{1,C}\!: r_{1,E} = 1\!:1$ and $r_{2,C}\!: r_{2,E} = 0\!:1$), an externally controlled trial (ECT) and an RCT design. *A1* indicates an analysis based on MSMs, and *A2* corresponds to an analysis via a permutation test. Source data are provided as a Source Data file.

The random-effects analysis (Table S3) and the operating characteristics in Figure 2 (for a fixed sample size n=75) showed that, for the EC-SCLC datasets, the available patient-level pre-treatment variables are insufficient to produce unbiased treatment effect estimates with adjustments in ECTs. Similarly, Figure S4 shows that the outcome distributions $\Pr(Y_i = 1|X_i, A_i = 0, S_i)$ of the IC ($S_i = 0$) and EC ($S_i = 1$) groups differ. For the Gales et al. study, the expected conditional outcomes of the IC ($S_i = 0$) tend to be larger than the expected conditional outcome in the EC group (CALGB-9732 and Picker et al studies). This inflates the type 1 error rates of the ECT. As expected, the type 1 error rate becomes more pronounced as the sample size of the ECT n increases.

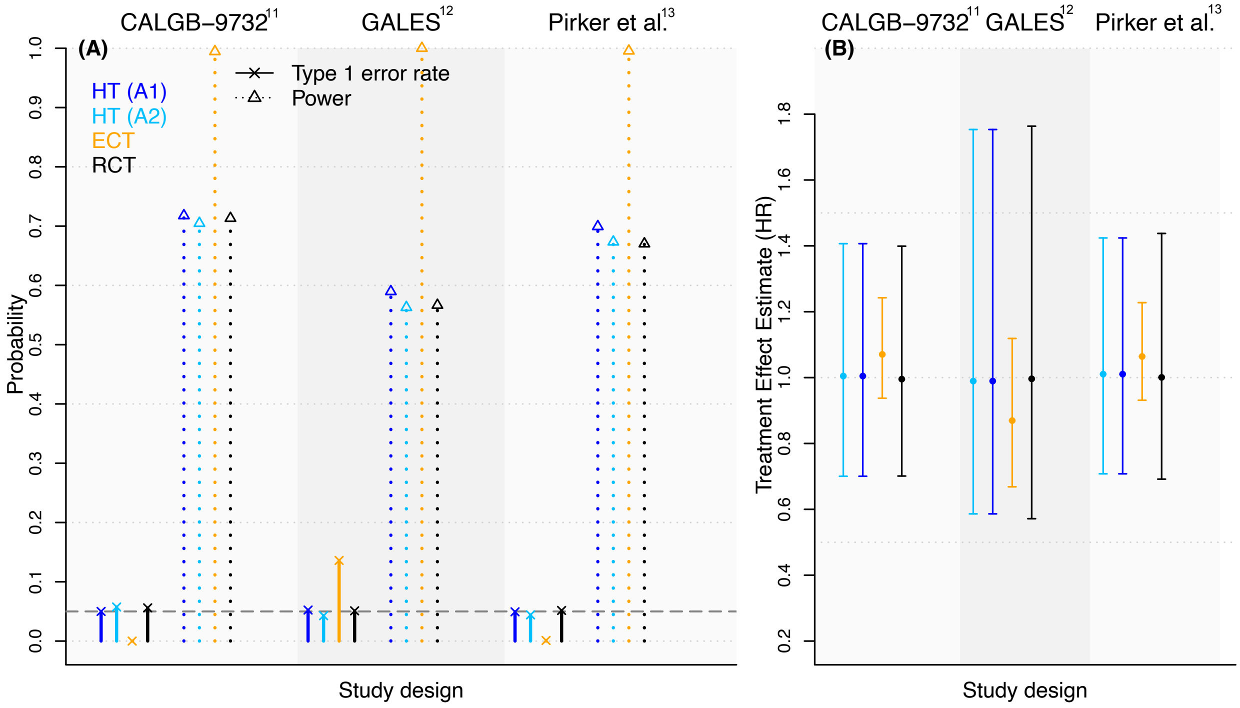

Supplementary Figure 5: Operating characteristics of different trial designs with overall survival as primary outcome (see Section 1.5). We consider a hybrid trial design (HR), an externally controlled trial (ECT) design and an RCT design. Panel A shows type I error rates (solid vertical lines with a cross on top) and power (dotted vertical lines with an arrow on top) across in 2,000 in silico trials (n=100). Panel B shows the variability/bias of treatment effect estimates (dots = average treatment effect estimates across in 2,000 in silico trials (n=100), vertical bars = 5th and 95th percentiles of across these in silico trials) based on the leave-one-study resampling algorithm. *A1* indicates an analysis based on MSMs, and *A2* corresponds to an analysis via permutation test. Source data are provided as a Source Data file.

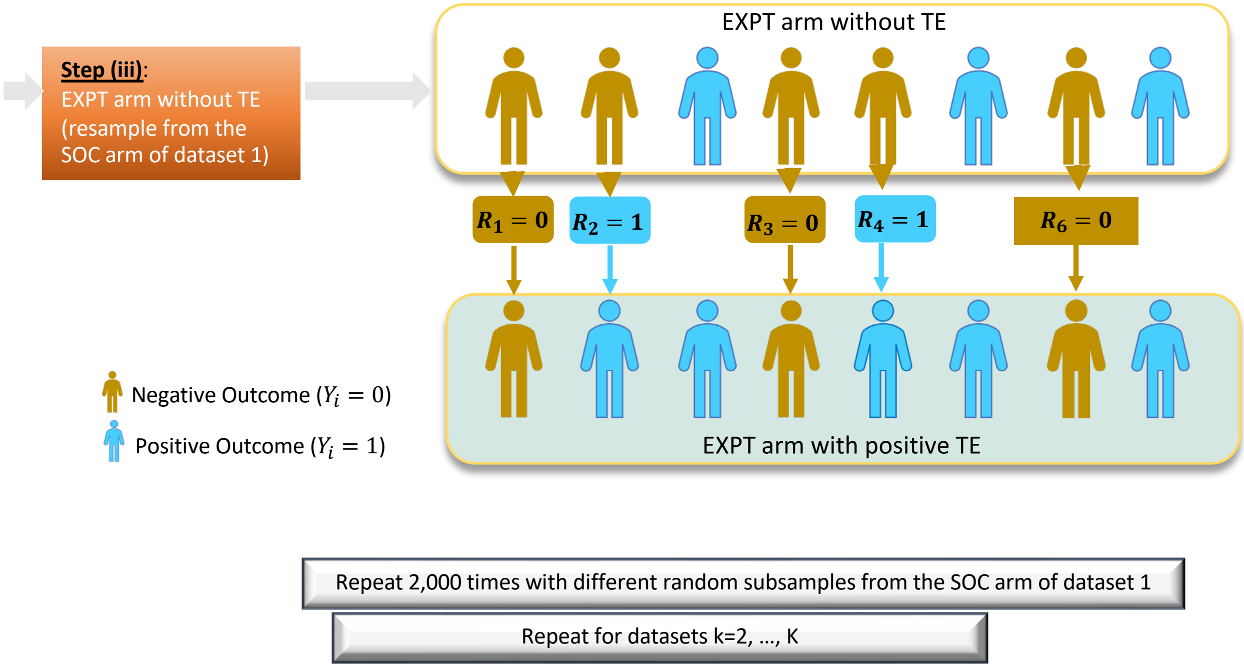

Supplementary Figure 6. Generating in silico trials with a positive treatment effect (TE). Graphical represenation of how we introduce a positive experimental treatment in the *leave-one-study-out resampling algorithm* (see Methods). For each patient on the *in silico* experimental treatment (EXPT) arm with negative response ($A_i = 1$, $Y_i = 0$), we randomly generate a binary random variable R_i, with $Pr(R_i = 1) = \pi$, representative of the treatment effect for patient i. If $R_i = 1$, then the negative outcome is relabeled as a positive outcome (i.e., we set $Y_i = 1$). If $R_i = 0$, then the outcome remains unchanged ($Y_i = 0$).

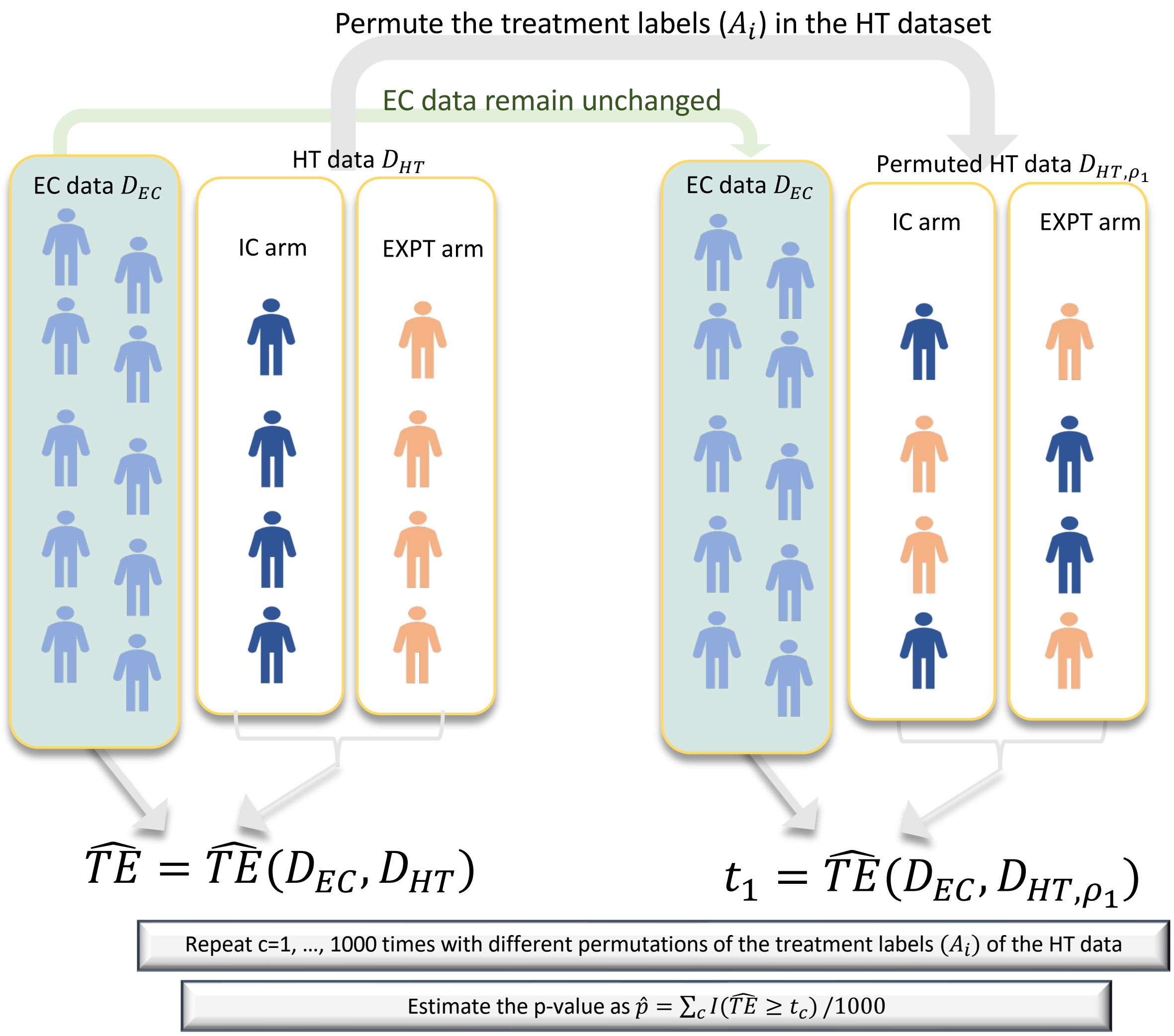

Supplementary Figure 7: A graphical representation of the permutation test (see Methods). Left panel: First, a treatment effects estimate $\widehat{TE} = \widehat{TE}(D_{HT}, D_{EC})$ is calculated using the hybrid trial (HT) data (D_{HT}) and the external control (EC) data (D_{EC}). Right panel: We randomly permute the treatment assignment variables $\{A_i\}_{i\leq n}$ in the HT ($A_{\rho_{\ell,1}}, A_{\rho_{\ell,2}} \ldots\ A_{\rho_{\ell,n}}$), while the assignment variables $\{A_i = 0\}_{i>n}$ in the EC dataset remain identical. We then compute the estimate $t_1 = \widehat{TE}\left(D_{HT,\rho_1}, D_{EC}\right)$ using the EC data (D_{EC}) and the permuted HT data (D_{HT,ρ_1}). Bottom part of the figure: We repeat the permutation step (Right panel) 1,000 times to obtain estimates $t_c, c = 1, \ldots, 1000$, using different random permutations of the treatment labels in the HT dataset. We then estimate the p-value as $\hat{p} = \sum_c I\left(\widehat{TE} \geq t_c\right)/1000$.

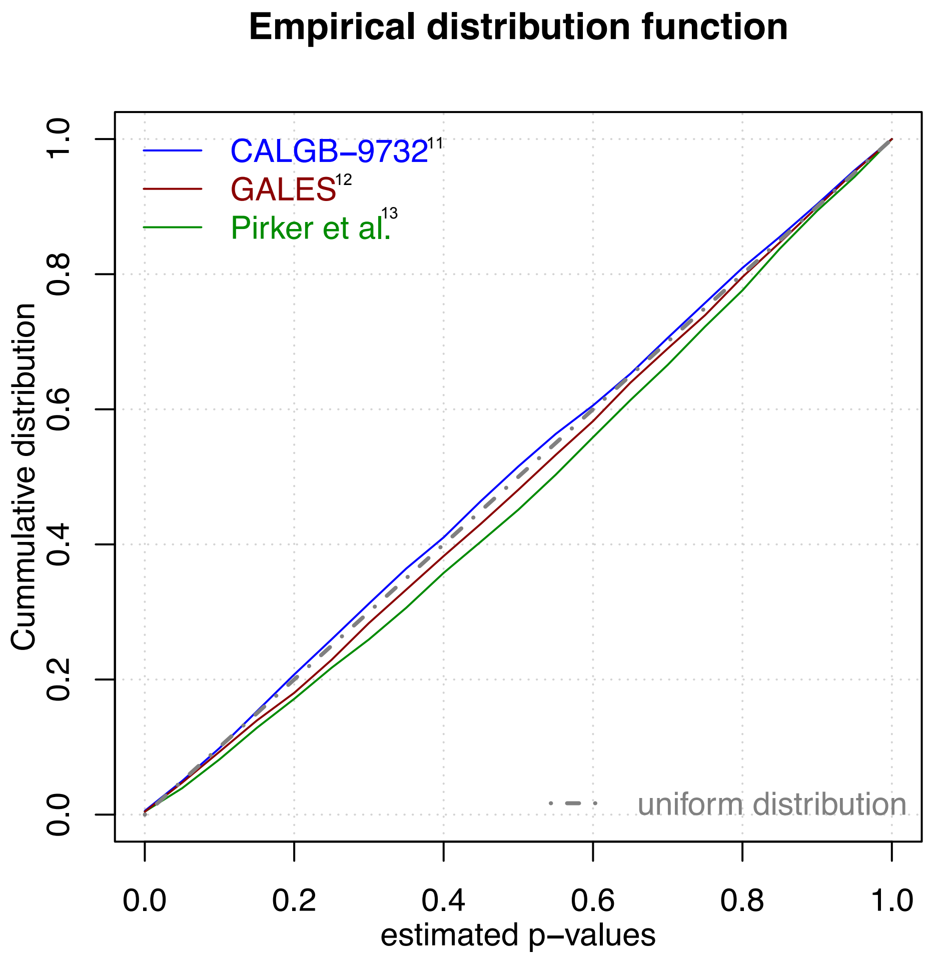

Supplementary Figure 8: The empirical distribution of the estimated p-values (without multiplicity adjustments) for the one-sided permutation test detailed in the Methods Section and Supplementary Figure 7. We used the *Leave-one-study-out resampling algorithm* to generate for each SCLC study 2,000 *in silico* trials (TE=0). Source data are provided as a Source Data file.

Supplementary References

1. Dobson AJ, Barnett AG. *An Introduction to Generalized Linear Models, Third Edition*.; 2008. doi:10.1080/02664760802695900
2. Self SG, Mauritsen RH, Ohara J. Power Calculations for Likelihood Ratio Tests in Generalized Linear Models. *Biometrics*. 1992;48(1):31-39. doi:10.2307/2532736
3. Agresti A. *An Introduction to Categorical Data Analysis: Second Edition*.; 2006. doi:10.1002/0470114754
4. Robins JM, Hernán MÁ, Brumback B. Marginal structural models and causal inference in epidemiology. *Epidemiology*. 2000;11(5):550-560. doi:10.1097/00001648-200009000-00011
5. Robins JM, Hernán MA. Estimation of the causal effects of time-varying exposures. In: *Longitudinal Data Analysis*. ; 2008; Chapter 23: 553-599. doi:10.1201/9781420011579.ch23
6. Robins J. A new approach to causal inference in mortality studies with a sustained exposure period-application to control of the healthy worker survivor effect. *Math Model*. 1986;7(9-12): 1393-1512. doi:10.1016/0270-0255(86)90088-6
7. Little RJ, D'Agostino R, Cohen ML, et al. The prevention and treatment of missing data in clinical trials. *N Engl J Med*. 2012;367(14):1355-1360. doi:10.1056/NEJMsr1203730
8. Ventz S, Comment L, Louv B, et al. The use of external control data for predictions and futility interim analyses in clinical trials. *Neuro Oncol*. 2022;24(2):247-256. doi:10.1093/neuonc/noab141
9. Collett D. *Modelling Survival Data in Medical Research, Third Edition*.; 2015.
10. Ventz S, Lai A, Cloughesy TF, Wen PY, Trippa L, Alexander BM. Design and Evaluation of an External Control Arm Using Prior Clinical Trials and Real-World Data. *Clin Cancer Res*. 2019;25(16):4993-5001. doi:10.1158/1078-0432.ccr-19-0820

11. Niell HB, Herndon JE, Miller AA, et al. Randomized phase III intergroup trial of etoposide and cisplatin with or without paclitaxel and granulocyte colony-stimulating factor in patients with extensive-stage small-cell lung cancer: Cancer and Leukemia Group B trial 9732. *J Clin Oncol*. 2005;23(16):3752-3759. doi:10.1200/JCO.2005.09.071

12. Thatcher N, Hirsch FR, Luft A V., et al. Necitumumab plus gemcitabine and cisplatin versus gemcitabine and cisplatin alone as first-line therapy in patients with stage IV squamous non-small-cell lung cancer (SQUIRE): An open-label, randomised, controlled phase 3 trial. *Lancet Oncol*. 2015;16(7):763-774. doi:10.1016/S1470-2045(15)00021-2

13. Pirker R, Ramlau RA, Schuette W, et al. Safety and efficacy of darbepoetin alfa in previously untreated extensive-stage small-cell lung cancer treated with platinum plus etoposide. *J Clin Oncol*. 2008;26(14):2342-2349. doi:10.1200/JCO.2007.15.0748

14. Salgia R, Stille JR, Weaver RW, McCleod M, Hamid O, Polzer J, Roberson S, Flynt A, Spigel DR. A randomized phase II study of LY2510924 and carboplatin/etoposide versus carboplatin/etoposide in extensive-disease small cell lung cancer. Lung Cancer. 2017 Mar;105:7-13. doi: 10.1016/j.lungcan.2016.12.020.

15. Ready NE, Pang HH, Gu L, Otterson GA, Thomas SP, Miller AA, Baggstrom M, Masters GA, Graziano SL, Crawford J, Bogart J, Vokes EE. Chemotherapy With or Without Maintenance Sunitinib for Untreated Extensive-Stage Small-Cell Lung Cancer: A Randomized, Double-Blind, Placebo-Controlled Phase II Study-CALGB 30504 (Alliance). J Clin Oncol. 2015 May 20;33(15):1660-5. doi: 10.1200/JCO.2014.57.3105.

16. Ventz S, Lai A, Cloughesy TF, Wen PY, Trippa L, Alexander BM. Design and Evaluation of an External Control Arm Using Prior Clinical Trials and Real-World Data. *Clin Cancer Res*. 2019;25(16):4993-5001. doi:10.1158/1078-0432.ccr-19-0820

17. Chinot OL, Wick W, Mason W, et al. Bevacizumab plus Radiotherapy–Temozolomide for Newly Diagnosed Glioblastoma. *N Engl J Med*. 2014;370(8):709-722.

doi:10.1056/NEJMoa1308345

18. Cho DY, Yang WK, Lee HC, et al. Adjuvant immunotherapy with whole-cell lysate dendritic cells vaccine for glioblastoma multiforme: A phase II clinical trial. *World Neurosurg*. 2012;77(5-6):736-744. doi:10.1016/j.wneu.2011.08.020
19. Lee EQ, Kaley TJ, Duda DG, et al. A multicenter, phase II, randomized, noncomparative clinical trial of radiation and temozolomide with or without vandetanib in newly diagnosed glioblastoma patients. *Clin Cancer Res*. 2015;21(16):3610-3618. doi:10.1158/1078-0432.CCR-14-3220
