# Supplementary material for: The design and evaluation of hybrid controlled trials that leverage external data and randomization
Source: Nat Commun. 2022 Oct 2;13:5783. doi: 10.1038/s41467-022-33192-1 (PMC9527257; doi:10.1038/s41467-022-33192-1)
Supplement: Supplementary file 2 — Description of Additional Supplementary Files [file 41467_2022_33192_MOESM2_ESM.pdf]

Supplementary Software 1: The file provides R Code to generate HT, ECT and RCT trials based on the design (HT, ECT, RCT) presented in the manuscript using either in silico data or data from completed trial (i.e. the ES-SCLC data). The R code also implements the leave-one-study-out cross validation resampling algorithm.
