# Supplementary material for: The design and evaluation of hybrid controlled trials that leverage external data and randomization
Source: Nat Commun. 2022 Oct 2;13:5783. doi: 10.1038/s41467-022-33192-1 (PMC9527257; doi:10.1038/s41467-022-33192-1)
Supplement: Supplementary file 4 — Reporting Summary [file 41467_2022_33192_MOESM4_ESM.pdf]

nature portfolio

Corresponding author(s): NCOMMS2035235B

Last updated by author(s): Aug 12, 2022

Reporting Summary

Nature Portfolio wishes to improve the reproducibility of the work that we publish. This form provides structure for consistency and transparency in reporting. For further information on Nature Portfolio policies, see our Editorial Policies and the Editorial Policy Checklist.

Statistics

For all statistical analyses, confirm that the following items are present in the figure legend, table legend, main text, or Methods section.

n/a	Confirmed	
☐	☒	The exact sample size (*n*) for each experimental group/condition, given as a discrete number and unit of measurement
☐	☒	A statement on whether measurements were taken from distinct samples or whether the same sample was measured repeatedly
☐	☒	The statistical test(s) used AND whether they are one- or two-sided<br>*Only common tests should be described solely by name; describe more complex techniques in the Methods section.*
☐	☒	A description of all covariates tested
☒	☐	A description of any assumptions or corrections, such as tests of normality and adjustment for multiple comparisons
☐	☒	A full description of the statistical parameters including central tendency (e.g. means) or other basic estimates (e.g. regression coefficient) AND variation (e.g. standard deviation) or associated estimates of uncertainty (e.g. confidence intervals)
☐	☒	For null hypothesis testing, the test statistic (e.g. *F*, *t*, *r*) with confidence intervals, effect sizes, degrees of freedom and *P* value noted<br>*Give P values as exact values whenever suitable.*
☒	☐	For Bayesian analysis, information on the choice of priors and Markov chain Monte Carlo settings
☒	☐	For hierarchical and complex designs, identification of the appropriate level for tests and full reporting of outcomes
☐	☒	Estimates of effect sizes (e.g. Cohen's *d*, Pearson's *r*), indicating how they were calculated

Our web collection on statistics for biologists contains articles on many of the points above.

Software and code

Policy information about availability of computer code

Data collection	We did not collect data, data were loaded into R via the standard R Base and MASS libraries.
Data analysis	Data analyses were conducted in R (version 4.0). We used the standard open source R libraries "Base", "MASS", "survival" and "sandwich", which implement the treatment-effect estimates/tests and the leave-one-study out algorithms. No customized software code is requires. R code used to generate the HT, ECT and RCT designs is provided via the Supplementary R code.

For manuscripts utilizing custom algorithms or software that are central to the research but not yet described in published literature, software must be made available to editors and reviewers. We strongly encourage code deposition in a community repository (e.g. GitHub). See the Nature Portfolio guidelines for submitting code & software for further information.

Data

Policy information about availability of data

All manuscripts must include a data availability statement. This statement should provide the following information, where applicable:

- Accession codes, unique identifiers, or web links for publicly available datasets
- A description of any restrictions on data availability
- For clinical datasets or third party data, please ensure that the statement adheres to our policy

The clinical trial datasets CALGB-973211 (NCT00003299), Pirker et al. (NCT00119613) and GALES25 used in our analyses are freely available for download from Project Data Sphere at https://data.projectdatasphere.org/. The GBM data were not generated for the purpose of this study, are protected and are not publicly available due to data privacy laws. Since restrictions apply to the availability of these data, please contact Drs Alexander (Brian_Alexander@dfci.harvard.edu), Wen

(Patrick_Wen@dfci.harvard.edu) and Rahman (RRAHMAN@BWH.HARVARD.EDU) for access to these data. De-identified patient level data (treatment outcomes and pre-treatment patient characteristics) will be shared upon reasonable request starting 1 months after publication for up to 3 years for research purposes. Model-based in silco data are provided via the supplementary R code.

Human research participants

Policy information about studies involving human research participants and Sex and Gender in Research.

Reporting on sex and gender	The sex of patients was determined by the investigators of the original studies. We conducted covariate-adjusted analysis, which included sex. Sex was not a factors that influenced the conclusions of our analyses.
Population characteristics	All relevant population characteristics of the study participants in each study is reported in Supplementary Tables 2 and 5.
Recruitment	We conducted secondary analysis using completed clinical trial data. Patient-recruitment was not conducted by ourself, but by the investigators of the clinical trails. We are not aware of any bias.
Ethics oversight	The study reported in this manuscript was approved by an IRB at the DFCI.

Note that full information on the approval of the study protocol must also be provided in the manuscript.

Field-specific reporting

Please select the one below that is the best fit for your research. If you are not sure, read the appropriate sections before making your selection.

☒ Life sciences ☐ Behavioural & social sciences ☐ Ecological, evolutionary & environmental sciences

For a reference copy of the document with all sections, see nature.com/documents/nr-reporting-summary-flat.pdf

Life sciences study design

All studies must disclose on these points even when the disclosure is negative.

Sample size	Sample size was determined to achieve a 80% power with a standard one-sided two-sample z-test for proportions with a 5% type I error rate .
Data exclusions	No data were excluded
Replication	We repeated the analysis in all available datasets using the re-sampling algorithm described in the manuscipt
Randomization	Block randomization 1:1
Blinding	All clinical studies used doubly blinded randomization

Reporting for specific materials, systems and methods

We require information from authors about some types of materials, experimental systems and methods used in many studies. Here, indicate whether each material, system or method listed is relevant to your study. If you are not sure if a list item applies to your research, read the appropriate section before selecting a response.

Materials & experimental systems

n/a	Involved in the study	
☒	☐	Antibodies
☒	☐	Eukaryotic cell lines
☒	☐	Palaeontology and archaeology
☒	☐	Animals and other organisms
☐	☒	Clinical data
☒	☐	Dual use research of concern

Methods

n/a	Involved in the study	
☒	☐	ChIP-seq
☒	☐	Flow cytometry
☒	☐	MRI-based neuroimaging

Clinical data

Policy information about clinical studies
All manuscripts should comply with the ICMJEguidelines for publication of clinical research and a completedCONSORT checklist must be included with all submissions.

Clinical trial registration	NCT00003299, NCT00119613, NCT00363415, NCT01439568, NCT00453154, PM24552318 PM25910950 PM22120301
Study protocol	Full study protocols are available from ClinicalTrials.gov and via the primary publication of these completed studies.
Data collection	Patients were recruited and data were collected between 1998-2018 in the US
Outcomes	We only conducted a single primary analysis for each of the studies (testing no OS treatment effect [TE] vs a positive TE).
